# Aberrant activation of the Hedgehog signalling pathway in squamous cell carcinoma of the vulva as a potential target for cancer therapy

**DOI:** 10.1038/s41598-021-96940-1

**Published:** 2021-09-03

**Authors:** Jason K. W. Yap, Khalil Uddin, Rachel Pounds, Danielle O’Neill, Sean Kehoe, Raji Ganesan, Christopher W. Dawson

**Affiliations:** 1 Birmingham Cancer Research UK Cancer Centre, Institute of Cancer and Genomic Sciences, University of Birmingham, Vincent Drive, Edgbaston, Birmingham, B15 2TT UK; 2 Pan Birmingham Gynaecological Cancer Centre, Birmingham City Hospital, Dudley Road, Birmingham, B18 7QH UK; 3 Birmingham Women’s NHS Foundation Trust, Mindelsohn Way, Edgbaston, Birmingham, B15 2TG UK; 4 Department of Microbiology & Infection, Warwick Medical School, University of Warwick, Coventry, CV4 7AL UK

**Keywords:** Cancer therapy, Gynaecological cancer, Oncogenes, Cancer, Oncology

## Abstract

In a previous study, we showed that the Hedgehog (Hh) signalling pathway is aberrantly activated in vulval squamous cell carcinoma (VSCC). In this study, we further validated our findings on a prospective cohort of primary VSCC cases, where immunohistochemical staining confirmed that key Hh pathway components were overexpressed in VSCC compared to normal vulval epithelium. We also undertook a series of in vitro studies to determine the extent of Hh pathway activation in VSCC-derived cell lines, and examine the consequences of pathway inhibition on the growth of these cells. We found that of six cell lines tested, four displayed elevated baseline Hh pathway activity that was dependent on SHH ligand, or in one case, a *PTCH1* gene mutation. Hh signalling appeared necessary to sustain cell growth, as SHH ligand depletion with Robotikinin or SMO inhibition, either with chemical inhibitors (Itraconazole or LDE-225) or SMO-specific siRNA, attenuated GLI1 activity and cell proliferation in both monolayer and organotypic raft culture. Furthermore, treatment of Hh-dependent cell lines with SMO inhibitors sensitised cells to Cisplatin. Findings from our study offer us the opportunity to explore further the development of targeted chemotherapy for women with VSCC driven by aberrant Hh activation.

## Introduction

Vulval cancer comprises 6% of all gynaecological malignancies reported in the UK, with squamous cell carcinoma (VSCC) contributing approximately 90% of cases. It is predominantly a disease of the elderly, with three-quarters of cases affecting those over 60-year-old^1^. While surgery is effective in managing low-volume localised tumours or metastatic disease to the groin, its role in advanced localised or systemic metastatic disease is usually limited to palliative setting for symptomatic control. Chemotherapy is rarely used as the sole therapy for VSCC but can be used as an adjunct to surgery or radiotherapy in advanced disease or for palliation^2^. Current chemotherapeutic regimes are not unique to VSCC and are based on those used for other gynaecological cancers. Owing to the rarity of the disease and the age of the affected population, who are generally unable to tolerate chemotherapy, experience on the use of chemotherapy in treating VSCC is limited to a small series of observational studies^2,3^. Unfortunately, the treatment response of VSCC to chemotherapies is variable, and toxicity remains a significant problem, rendering it unsuitable for most patients. As such, there is a need to develop better targeted medical therapies with lower toxicity.

To date, only a small number of studies have utilised molecular profiling to identify dysregulated cell signalling pathways which might constitute potential therapeutic targets in VSCC. While frequent mutations in the Ras and PI3 kinase-mTOR-PTEN pathways have been described^4–8^, we recently found evidence of aberrant Hedgehog (Hh) pathway activation in VSCC^9^. Hh pathway dysregulation is not unique to VSCC, as it has been described in a variety of cancers, including cervical and ovarian cancer^10,11^. The Hh pathway regulates morphogenesis and patterning during embryogenesis. After that, it maintains some epithelial stem cell populations and is activated during tissue regeneration and wound healing. However, dysregulation of this pathway has been described in cancers at several sites^12–14^. Hh signalling is initiated by binding of a Hh ligand (Sonic Hh, Indian Hh or Desert Hh) to PTCH1. This transmembrane protein represses the activity of SMO, a G protein-coupled receptor. In response to Hh ligand binding, PTCH1 repression of SMO is relieved, and the activity of three GLI transcription factors is induced. While GLI1 is a transcriptional activator and GLI3, a transcriptional repressor; GLI2 can function as both. The importance of Hh signalling in carcinogenesis flows from its activation of a variety of downstream effectors, which impact on cell growth, survival, differentiation, angiogenesis and cell motility. Dysregulation of Hh signalling may follow overexpression of HH ligand, loss-of-function in PTCH1, gain-of-function mutations in SMO and epigenetic modulation of pathway components^12–14^.

In our original study, we reported frequent overexpression of key Hh pathway components in > 90% of VSCC cases and posited that the Hh pathway was aberrantly activated through a mechanism involving overexpression of SHH ligand^9^. From a clinical viewpoint, our study revealed that tumours displaying a weak expression of PTCH1, on a background of increased Hh pathway activity, were more likely to give rise to local disease recurrence following surgery, advocating the use of PTCH1 immunostaining as a diagnostic biomarker to predict the likelihood of local recurrence. As the Hh pathway usually is “switched off” or only transiently activated in most tissues in adulthood, our findings offer the opportunity to explore the clinical efficacy of Hh inhibitors as a new targeted therapy for VSCC, either alone, or in combination with current chemotherapy regimens; especially in those with locally advanced or metastatic disease where surgery has a limited role^2^.

To this end, we undertook a preclinical study to determine whether Hh pathway inhibitors can be used to treat VSCC. Using tumour tissues obtained from our prospective cohort of patients with primary VSCC, we evaluated the expression of key Hh pathway components, comparing them to normal vulval epithelium from age-matched controls. Given that the Hh pathway is often activated in response to inflammation and tissue remodelling, we set out to determine the extent of pathway activation in a panel of established VSCC lines in vitro, and evaluate the effects of two FDA-approved SMO inhibitors, LDE-225 and Itraconazole, on the growth of VSCC cell lines both in monolayer and 3D organotypic raft culture.

## Results

### Key Hedgehog pathway components are over-expressed in VSCC compared to age-matched normal vulval epithelium

**Figure 1:**
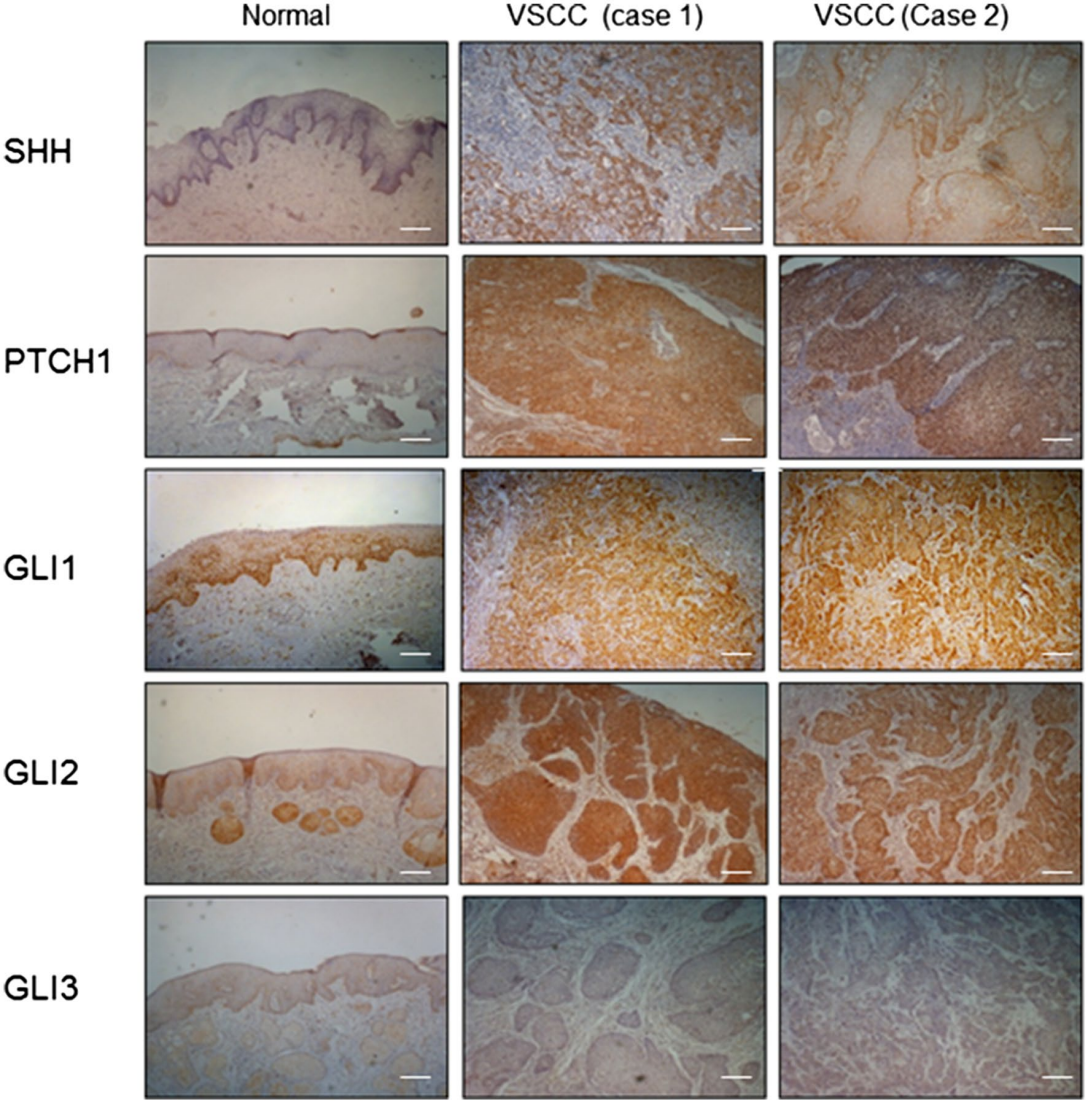
Key components of the Hedgehog pathway are overexpressed in two cases of primary VSCC compared to age-matched normal vulval epithelium. Representative Immunohistochemical staining showing differential expression of SHH, PTCH1, GLI1, GLI2 and GLI3 in normal vulval squamous epithelium and two cases of primary VSCC (original magnification × 200). Bar = 50 μm.

To consolidate the findings of our previous study, IHC staining was performed on a cohort of 49 primary VSCC tumour biopsies and 15 age-matched normal vulval epithelial tissue specimens, to evaluate expression of key Hh pathway components (SHH, PTCH1, GLI1, GLI2 and GLI3); the latter obtained from patients undergoing vulva/perineal surgery unrelated to pre-malignant or malignant disease. Patients with primary VSCC were treated with radical excision and none had received adjuvant chemo- or radiotherapy. In both tumour and normal vulval epithelium, expression of SHH ligand was localised to the cytosol; PTCH1 to the membrane, cytosol and nucleus; and GLI1, GLI2 and GLI3 to the cytosol and nucleus (Fig. 1). Insufficient tissue material was available for 10 cases of VSCC staining for SHH; 9 cases of VSCC for PTCH1; 1 case of normal vulval epithelium for GLI1 and GLI2; 10 cases of VSCC and 3 cases for normal vulval epithelium for GLI3.

In agreement with our previously published finding, expression of SHH ligand was extremely weak in normal vulval epithelium (median 0) and, when expressed, was restricted to the cytosol in basal epithelial cells. Similarly, very low levels of PTCH1 (median 0.5), GLI1 and GLI2 (median 1.75) were observed in the basal cell layer of normal epithelium, with expression of the GLI1 and GLI2 proteins largely restricted to the nucleus and cytosol. In contrast, these key Hh pathway components were found to be significantly over-expressed in VSCC compared to normal vulval epithelium: SHH (median 1.0), PTCH1 (median 2.0), GLI1 (median 3.5) and GLI2 (median 3.5), and their expression were uniformly distributed across the tumour (Table 1). Although expression of the negative Hh pathway regulator, GLI3, was higher in normal vulval epithelium compared to that in the tumour, this was not statistically significant.

**Table 1:** Summary of Hedgehog pathway component expression in VSCC and age-matched normal vulval epithelium.

Hh pathway components	Normal vulva epithelium vs. VSCC	Number (n)	Median score (range)	Wilcoxon two sample test
SHH	Normal vulva	15	0 (0–0)	*p < 0.0001
	VSCC	39	1 (0–6)	
PTCH1	Normal vulva	15	0.5 (0–1)	*p < 0.0001
	VSCC	40	2 (0.5–6)	
GLI1	Normal vulva	14	1.75 (0–2.5)	
	VSCC	49	3.5 (0–8)	*p < 0.0001
GLI2	Normal vulva	14	1.75 (0–2.5)	*p < 0.0001
	VSCC	49	3.5 (0–8)	
GLI3	Normal vulva	12	2 (0–3)	*p* = 0.1201
	VSCC	39	1 (0–3)	

*p < 0.05, Wilcoxon two sample test indicates that the difference in the Allred score was significant.

### Hedgehog pathway activity is elevated in four of six VSCC-derived cell lines

**Figure 2:**
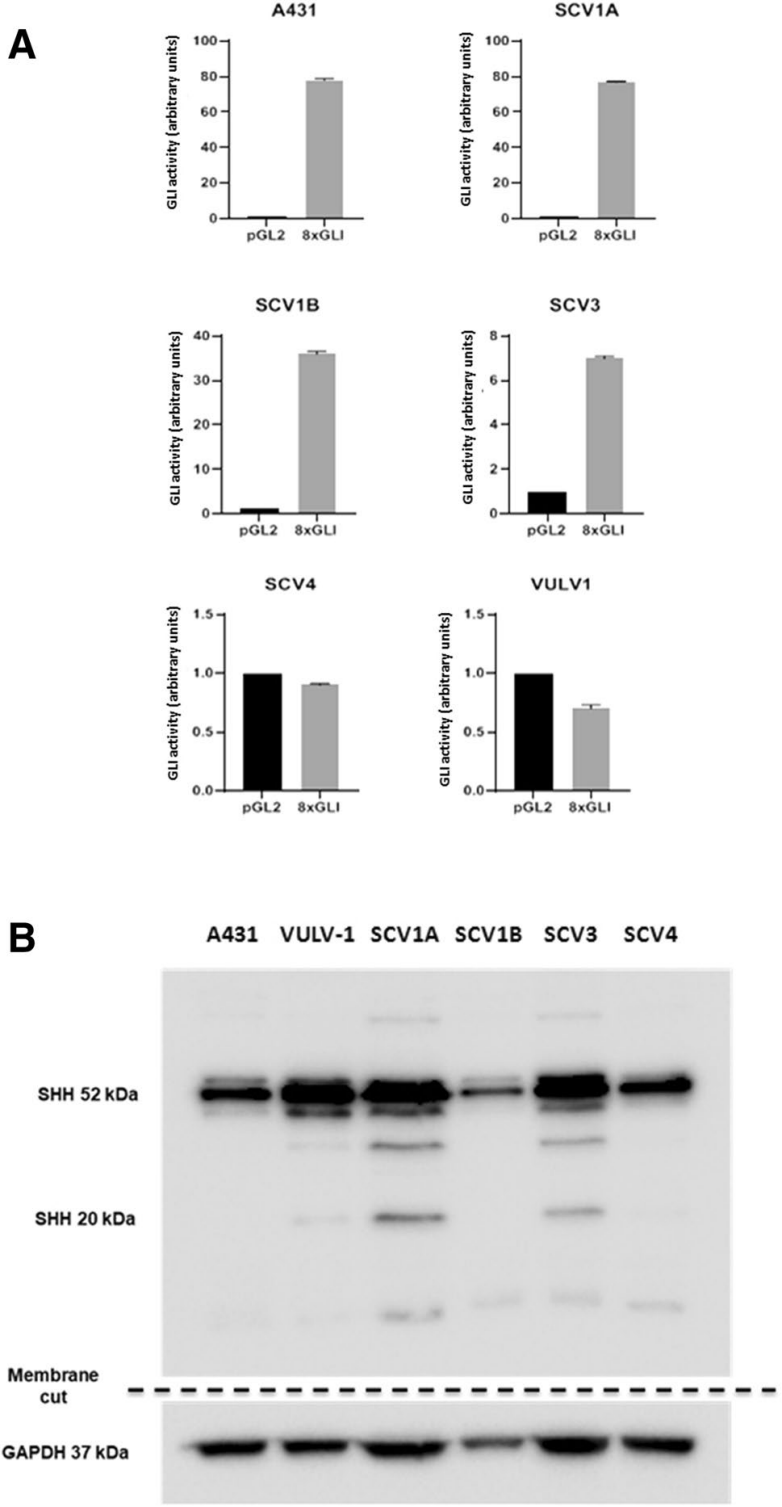
Baseline Hh pathway activity is elevated in 4 of 6 VSCC-derived cell lines. (**A**) Transient transfection of cells with a GLI-reporter (pGLIx8) revealed elevated baseline Hh-GLI activity in A431, UM‐SCV‐1A, UM‐SCV‐1B and UM‐SCV‐3 but not in UM‐SCV‐4 and UCI-VULV1. (**B**) Representative western blotting analysis demonstrating expression of mature, “processed” SHH protein in UM-SCV1A, UM-SCV1B and UM-SCV3; low levels were observed in UM-SCV4, A431 and UCI-VULV1 cells. All experiments were performed three times.

Given that key Hh pathway components were found to be overexpressed in VSCC tissue specimens, we set out to investigate whether cell lines derived from VSCC tissue retained elevated pathway activity in vitro. A small panel of VSCC cell lines were obtained, one of which, A431, is known to harbour a mutation in the *PTCH1* gene; the latter resulting in constitutive Hh pathway activation in the absence of SHH ligand^15^. Transient GLI-luciferase reporter assays revealed elevated baseline Hh-GLI activity in 4 out of 6 cell lines (Fig. 2A). A431, along with UM‐SCV‐1A, UM‐SCV‐1B and UM‐SCV‐3 displayed high “baseline” levels of GLI activity, whereas UM‐SCV‐4 and UCI-VULV-1 showed no activity (Fig. 2A). Western blotting analysis (Fig. 2B) confirmed expression of processed/mature forms of SHH protein in UM-SCV1A and UM-SCV3, lower levels in UM-SCV1B and SCV4, and very low levels in VULV1 and A431 cells. Further qPCR and Western blot analysis revealed that all the Hh components were upregulated in VSCC cell lines compared to NVK strains, whereas GLI3, a negative regulator of the Hh pathway, was expressed at high levels in three NVK strains, and virtually absent from the VSCC cell lines (Supplementary Fig. 2). While the baseline levels of GLI2 proteins are broadly comparable between cell lines, the baseline level of GLI1 appears to be higher in cell lines displaying elevated GLI-reporter activity, except for UM-SCV1B. Our analysis revealed a correlation between SHH expression and GLI-reporter activity in the VSCC cell lines, indicating that SHH overexpression may constitute the mechanism responsible for Hh pathway activation in these VSCC-derived cell lines. RNA was isolated from cells and RT-PCR used to measure the levels of SHH ligand in the cell lines; normal vulval keratinocytes (NVK) were included as controls. Components of the Hh pathway expressed in panel of VSCC cell lines and NVK were demonstrated in Supplementary Fig. 1. In general, NVK lacks expression of Hh pathway components when compared to VSCC cell lines, except for GLI3, a negative Hh regulator. Interestingly, the levels of SHH mRNA were also elevated in A431 cells, even though this cell line harbours a mutation in the *PTCH1* gene and, as such, do not require SHH ligand for Hh pathway activation.

### The growth of Hh-dependent VSCC cell lines is mediated through the Canonical Hedgehog signalling pathway and is dependent on SHH ligand

**Figure 3:**
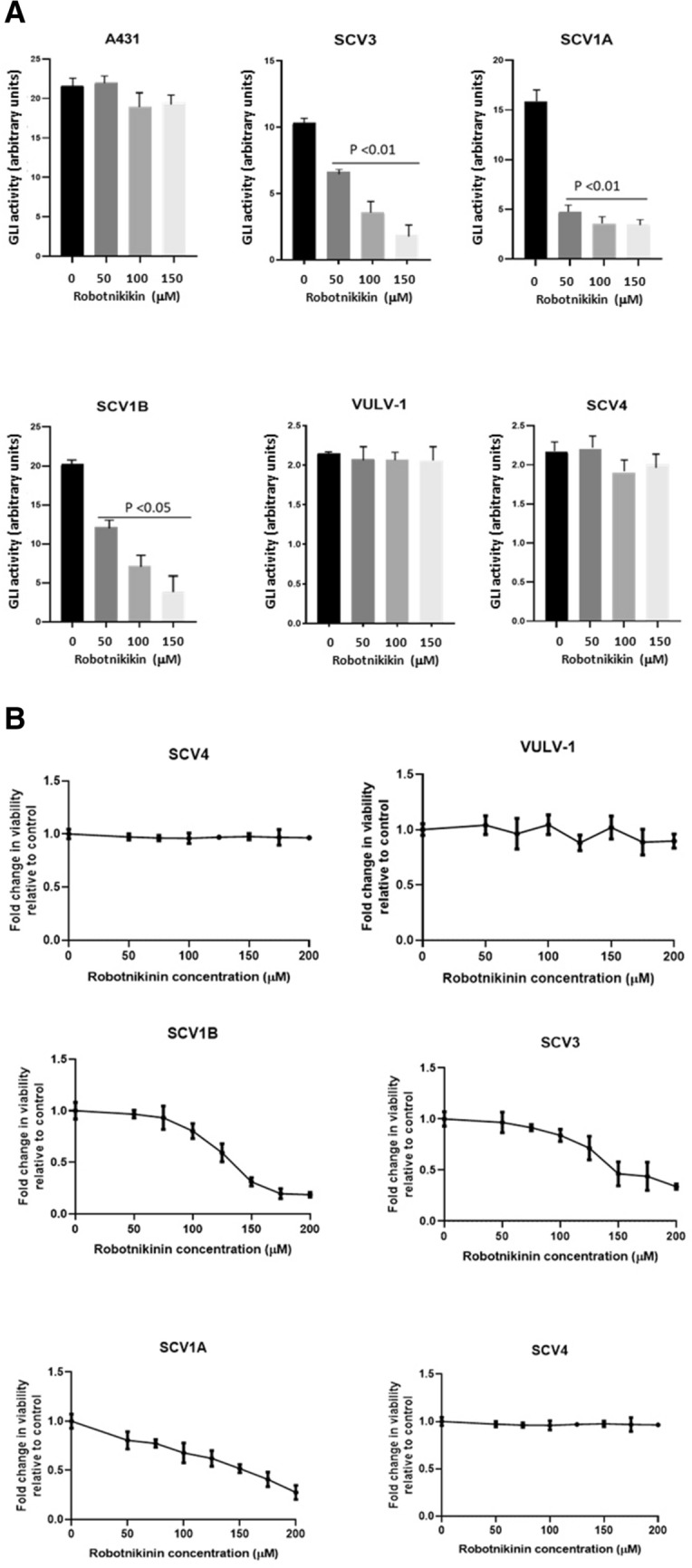
Treatment of Hh-dependent VSCC cells with the SHH antagonist, Robotikinin, attenuates GLI-reporter activity and cell proliferation. (**A**) Treatment of VSCC cell lines with the SHH antagonist, Robotikinin, led to a significant reduction in GLI-luciferase reporter activity in UM‐SCV‐1A, UM‐SCV‐1B and SCV3, but had no effect on A431, or the low baseline GLI-reporter activity in UM-SCV4 and UCI-VULV-1. (**B**) XTT assays showing a dose-dependent reduction in cell proliferation in Hh-dependent (UM-SCV-1A, UM-SCV1B, SCV3) but not Hh-independent (SCV-4, UCI-VULV1) VSCC cell lines treated with Robotikinin; Robotikinin had no significant effects on A431 cells.

The strong association between SHH ligand expression and GLI-reporter activity prompted us to establish whether Hh signalling in these cells was SHH ligand-dependent. We made use of Robotnikinin, an SHH antagonist, which binds and inactivates SHH ligand^16^ to treat Hh-dependent and Hh-independent lines. GLI-reporter activity and cell proliferation were then measured using GLI-reporter assay and XTT cell proliferation assay, respectively. Cells were transiently transfected with a GLI-reporter plasmid prior to treatment with increasing doses of Robotnikinin. While Robotnikinin did not affect GLI-reporter activity in the Hh-independent lines, UM-SCV-4 and UCI-VULV-1, treatment of the Hh-dependent cell lines, UM-SCV-1A, UM-SCV-1B and UM-SCV-3 cells, led to a significant reduction of GLI-reporter activity (Fig. 3A), indicating that the Hh pathway in these cells is driven by SHH ligand.

Interestingly, treatment of A431 cells, which harbours a *PTCH1* gene mutation, did not affect baseline GLI-reporter activity, confirming that A431 cells do not require SHH ligand to activate the Hh pathway. In keeping with its impact on Hh/GLI activity, Robotnikinin treatment attenuated cell proliferation in a dose-dependent manner in Hh-dependent lines but not the Hh-independent lines or A431 (Fig. 3B). Collectively, our results suggest that aberrant activation of Hh pathway in VSCC is driven via the canonical pathway either by *PTCH1* gene mutation, as seen in A431, or through autocrine expression of SHH ligand, as observed in the UM-SCV-1A, UM-SCV-1B, and UM-SCV-3 cell lines.

### Treatment of Hh-dependent VSCC cells with Hh pathway inhibitors, attenuates GLI-reporter activity, leading to cell growth inhibition

Building on this observation, we decided to investigate the biological consequence of inhibiting the Hh pathway in VSCC lines with two FDA-approved Hh pathway inhibitors, Itraconazole and LDE-225, both of which inhibit the canonical Hh signalling by binding to and inactivating SMO^17,18^. The aim here was to establish if clinically available Hh inhibitors could be used in the clinical setting to treat VSCC.

**Figure 4:**
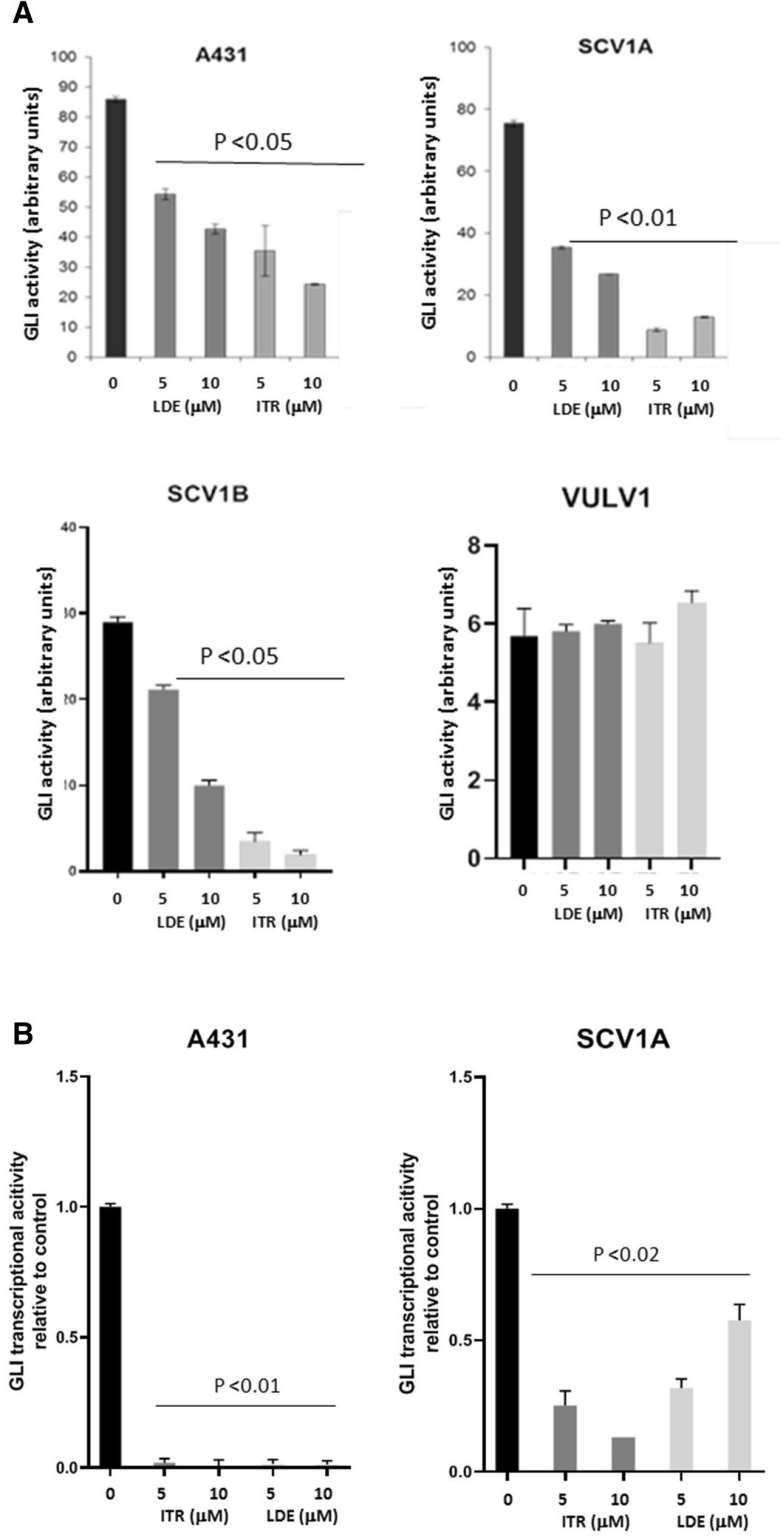

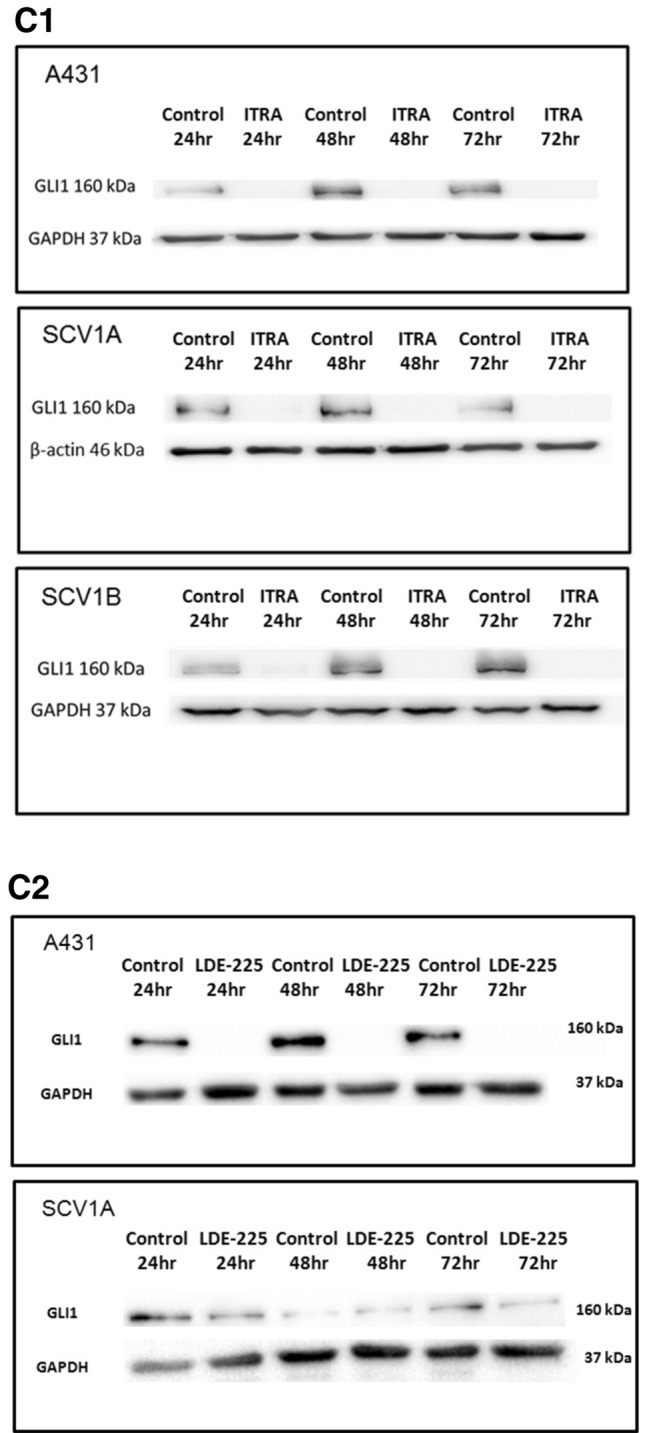

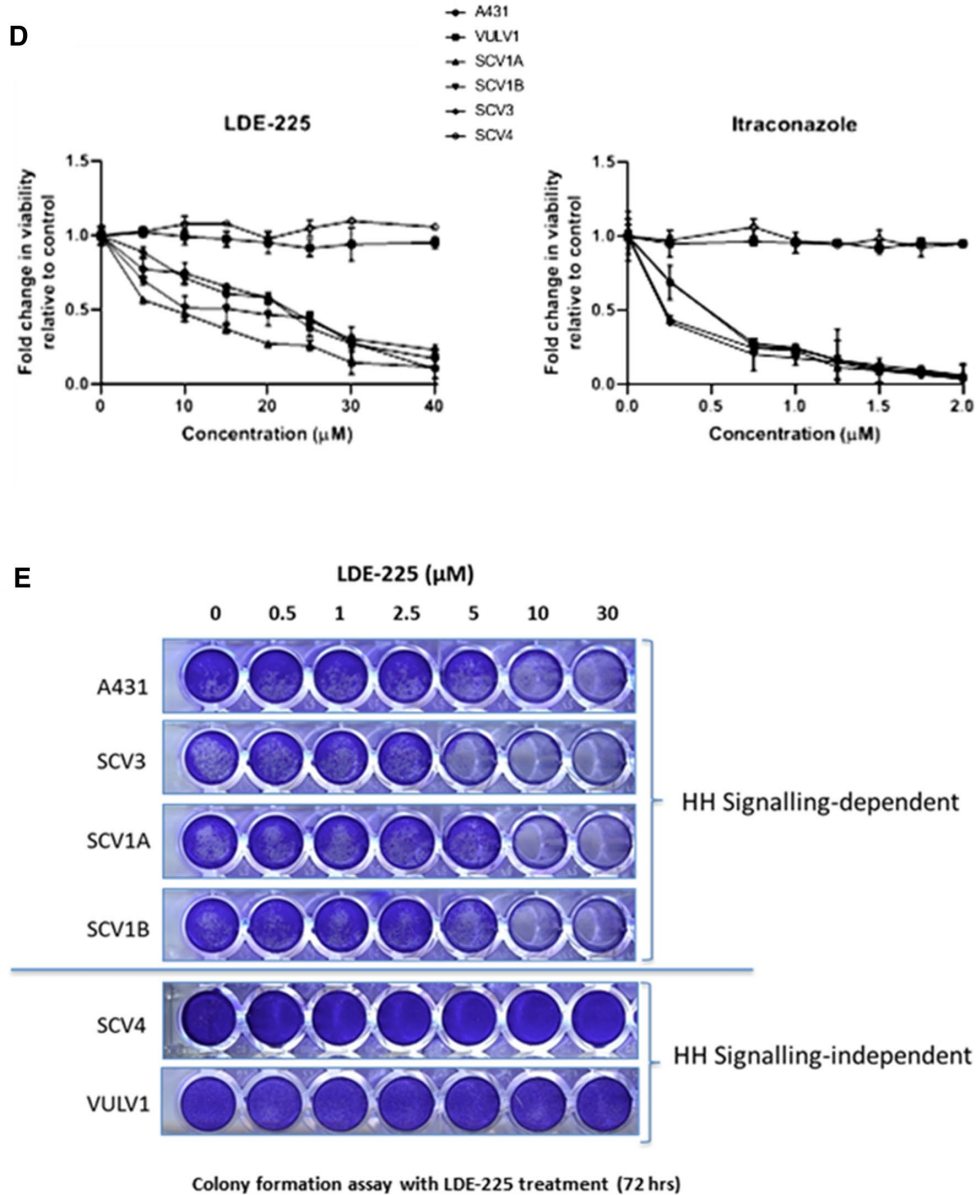
Treatment of Hh-dependent VSCC cells with SMO inhibitors, LDE-225 and Itraconazole, attenuates GLI-reporter activity and cell proliferation. (**A**) Treatment of VSCC cell lines with the SMO inhibitors, Itraconazole (ITR) or LDE-225, led to a significant reduction in GLI-luciferase reporter activity in A431, UM‐SCV‐1A and UM‐SCV‐1B, but had no effect on the low baseline GLI-reporter activity in UCI-VULV1. (**B**) Itraconazole (ITR), and LDE-225 (LDE), significantly reduced expression of GLI1 mRNA in UM-SCV1A and A431 cells 48 h after treatment. (**C**). Western blot showing the reduction in GLI1 expression in Hh-responsive cells (A431, UM-SCV1A, UM-SCV1B) treated with (**C.1**) 10 µM Itraconazole or (**C.2**) 25 µM LDE-225 for the indicated times (See Supplementary Fig. 3A,B for raw data). (**D**) XTT assay showing a dose-dependent reduction in cell proliferation in Hh-dependent (A431, UM-SCV1A, UM-SCV1B, SCV3) but not Hh-independent (SCV4, UCI-VULV1) VSCC cell lines when treated with Itraconazole or LDE-225 for 72 h. (**E**) Short-term cell viability assay demonstrating a significant decrease in cell proliferation in Hh-dependent VSCC cell lines (A431, UM-SCV1A, UM-SCV1B, SCV3) but not in Hh-independent cell lines (SCV4, UCI-VULV1) when treated with LDE-225 for 72 h. Samples used for western blotting analysis were derived from the same experiment and gels/blots were processed in parallel. All experiments were performed three times.

Firstly, we evaluated the effects of the SMO inhibitors on GLI-activity. VSCC cell lines were transiently transfected with a GLI-reporter plasmid prior to treatment with 5 µM and 10 µM LDE-225 or Itraconazole for 48 h. As shown in Fig. 4A, treatment of A431, UM‐SCV‐1A and UM‐SCV‐1B, with both SMO inhibitors led to a significant reduction in GLI-reporter activity, confirming that Hh pathway activation in these cell lines was SMO dependent. As expected, both Itraconazole and LDE-225 had no effect on the low baseline levels of GLI activity in UCI-VULV1 (Fig. 4A). In agreement with the GLI-reporter assays, q-PCR analysis showed that the expression of GLI1 mRNA was reduced in the Hh-dependent A431 and UM‐SCV‐1A cell lines treated with 5 µM or 10 µM 48 h post treatment (Fig. 4B). Western blotting analysis also confirmed a reduction in GLI1 protein in the Hh-dependent A431, UM‐SCV‐1A and UM‐SCV‐1B cell lines 24 h after treatment with 10 µM Itraconazole, which continued to remain low 48 and 72 h following treatment (Fig. 4C.1, Supplementary Fig. 3A). Similarly, down regulation of the GLI1 protein was also observed with 25 µM LDE-225 treatment (Fig. 4C.2, Supplementary Fig. 3B). Similar findings were observed for GLI2 (Supplementary Fig. 4), where treatment of UM-SCV1A, UM-SCV1B and A431 cells with either Itraconazole or LDE-225 for 24 and 48 h respectively, resulted in a robust reduction in GLI2 protein expression.

We then evaluated the consequence of attenuating Hh pathway activity on cell growth, by treating cells with increasing concentrations of LDE-225 or Itraconazole. All 6 VSCC cell lines were treated with escalating doses of Itraconazole or LDE-225 and, after 72 h, cell proliferation measured by XTT and colony formation assay. Figure 4D shows that treatment with Itraconazole or LDE-225 reduced cell proliferation in a dose-dependent fashion in Hh-dependent cell lines, A431, UM‐SCV‐1A, UM‐SCV‐1B and UM‐SCV‐3 but had little effect on cell proliferation in the Hh-independent cell lines, UM‐SCV‐4 and UCI-VULV1. Similarly, a short-term cell viability assay demonstrated that cell growth/viability was inhibited in a dose-dependent manner in Hh-dependent cell lines and not in Hh-independent cell lines treated with increasing doses of LDE-225 (Fig. 4E). Collectively, our results show that 4 of the 6 VSCC-derived cell lines were reliant on Hh-pathway activity for cell proliferation, as SMO inhibitors attenuate expression of GLI1 and cell proliferation.

### SMO knockdown in the Hh-responsive VSCC cell line, UM‐SCV‐1B, inhibits GLI1 activity and cell proliferation

**Figure 5:**
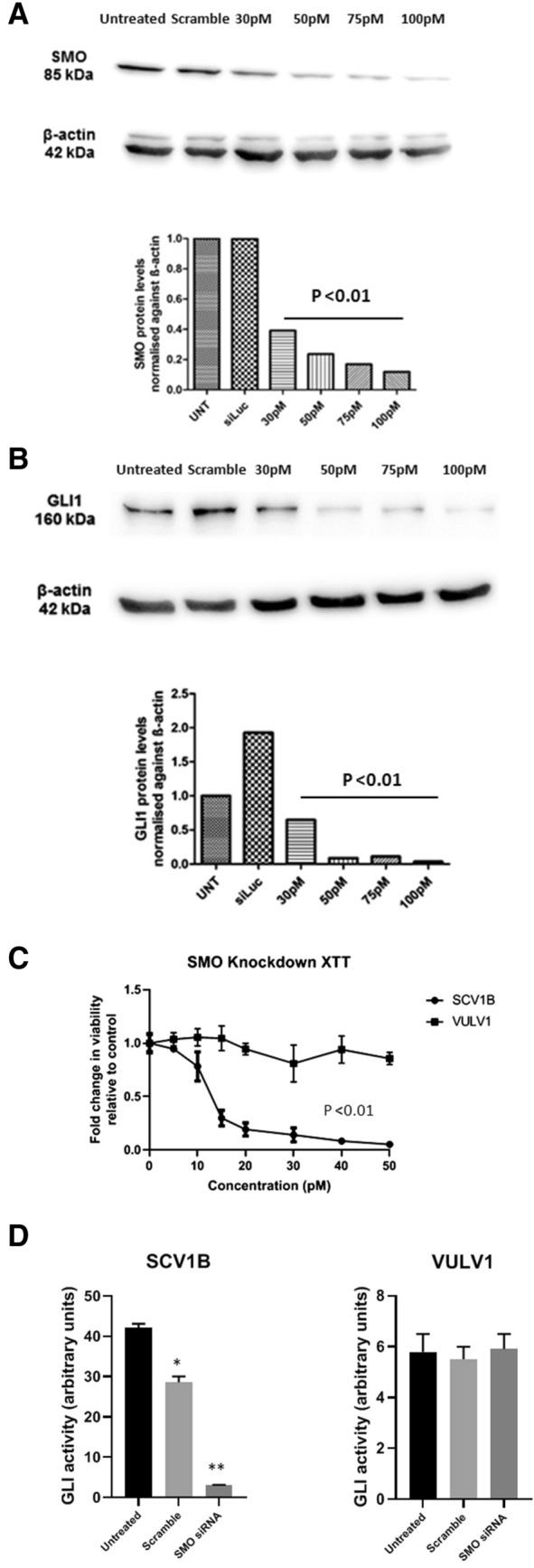
Transient transfection of Hh-responsive VSCC cell line, UM‐SCV-1B, with SMO-specific siRNA (HSS185995), reduced GLI1 expression and cell proliferation. Western blotting analysis showing downregulation of (**A**) SMO protein and (**B**) a concomitant reduction in the expression of GLI1 protein, following transfection with SMO-specific siRNAs. No effect on the expression of SMO or GLI1 was observed with a control “scrambled” siRNA (see Supplementary Fig. 5 for raw data). (**C**) Transient transfection of a SMO-siRNA into the Hh-responsive UM‐SCV‐1B cell line resulted in a dose-dependent reduction in cell viability, while having little or any effect on the Hh-independent UCI-VULV1 cell line. (**D**) GLI-luciferase reporter assays demonstrating the ability of two independent SMO-specific siRNAs to attenuates Hh/GLI signalling in the Hh-dependent UM-SCV-1B cell line but not the Hh-independent UCI-VULV1 cell line. Samples for WB were derived from the same experiment and gels/blots were processed in parallel. All experiments were performed three times. *p < 0.05, *p* < 0.005, NS = none significant.

To ensure that the effects of SMO inhibitor treatment were not due to “off-target” effects, representative VSCC cell lines were transiently transfected with SMO-specific siRNAs, and both GLI activity and cell proliferation examined. Firstly, the UM‐SCV‐1B cell line was transiently transfected with 3 different concentrations of a SMO-specific siRNA (siRNA-SMO-3) and a scrambled siRNA negative control and protein lysates generated after 48 h. Western blotting analysis confirmed downregulation of SMO protein expression and a concomitant reduction in expression of GLI1, following transfection with SMO-specific siRNAs (Fig. 5A,B, Supplementary Fig. 5). No effect on the expression of SMO or GLI1 was observed with the negative control siRNA, confirming the specificity of the SMO-siRNA (Fig. 5A,B). Transient transfection of SMO-siRNA into the Hh-responsive UM‐SCV‐1B cell line, resulted in an inhibition of cell in a dose-dependent manner (Fig. 5C). Conversely, transient transfection of SMO-siRNA in UCI-VULV1 had little or no effect on cell viability. To confirm the specificity, an additional SMO-specific siRNA was obtained and, along with the original SMO siRNA, transfected into UM‐SCV‐1B and UCI-VULV1 cells along with the GLI-Luc reporter. GLI reporter activity was attenuated in UM-SCV-1B with SMO siRNA, whereas the scrambled siRNA had only marginal effects on reporter activity. In marked contrast, SMO-specific siRNA had no effect on GLI reporter activity in UCI-VULV1 (Fig. 5D). All experiments were repeated using a different SMO siRNAs and the results again showed attenuation of GLI reporter activity and growth inhibition of UM-SCV-1B but not UCI-VULV1 cells (Supplementary Fig. 6A,B,C). Collectively, our data further reaffirm that inhibition of SMO leads to the attenuation of GLI1 activity and growth inhibition, an effect that is only specific to cells that are reliant on Hh pathway activity.

### SMO inhibitors sensitise Hh-dependent VSCC-derived cell lines to Cisplatin.

**Figure 6:**
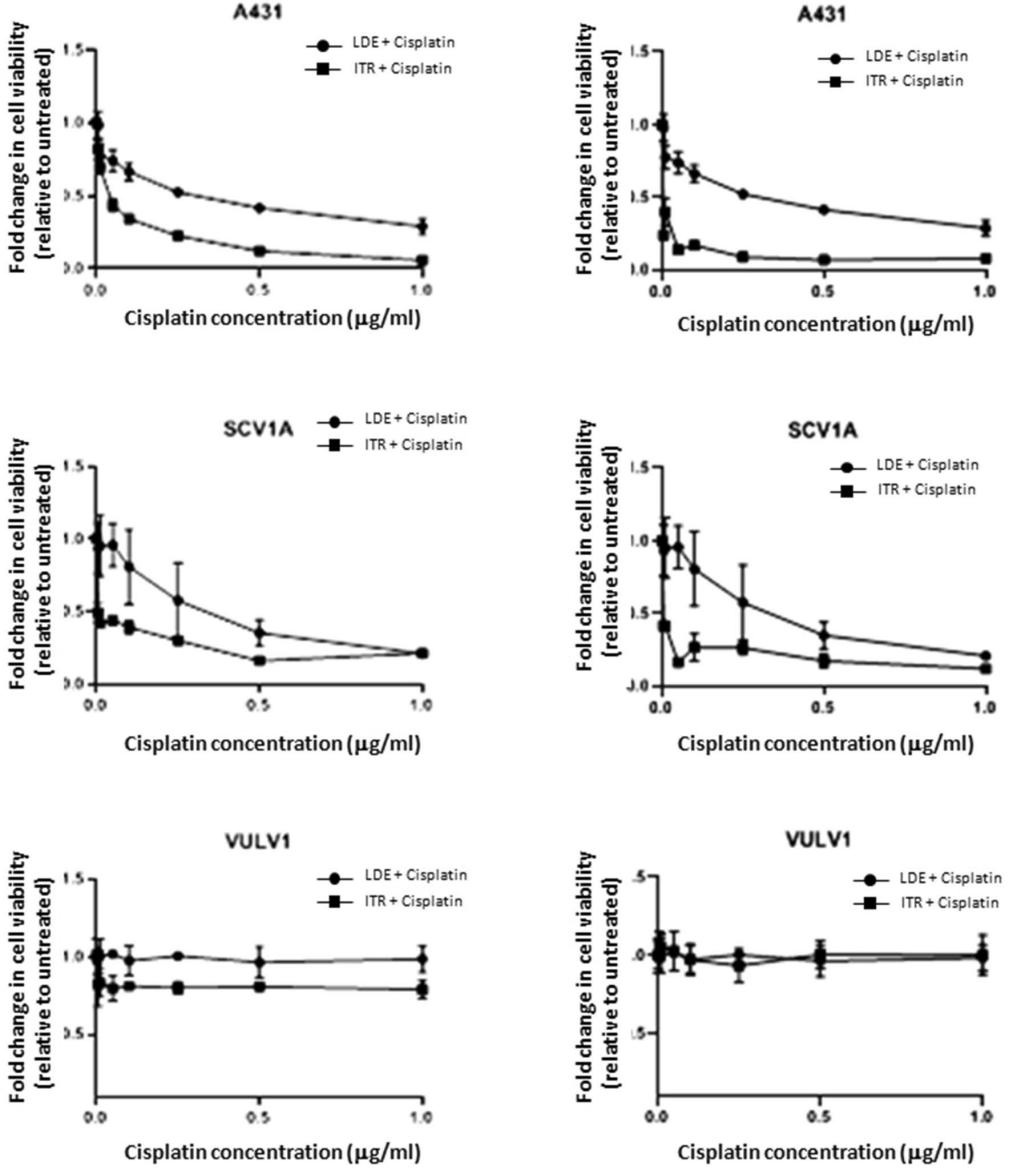
The SMO inhibitors, LDE-225 and Itraconazole, sensitise Hh-dependent VSCC-derived cell lines to Cisplatin. Co-treatment of Hh-dependent VSCC cell lines with LDE-225 or Itraconazole at IC50 dose and escalating dose Cisplatin leads to a profound leftward shift of the cell viability curve, indicating that Hh-inhibitors sensitise these cells to Cisplatin, but this effect was not observed in Hh-independent cell, UCI-VULV1. All experiments were performed three times.

Aberrant expression of the Hh pathway has been shown to protect cancer cells against chemotherapeutic agents. To establish whether Hh inhibitors could sensitise cell lines to Cisplatin, cells were treated with increasing doses of the drug in the presence or absence of LDE-225 or Itraconazole. Cell viability was measured by XTT assay 72 h post treatment. The results of the combined therapy revealed that the IC50 of Cisplatin was reduced from 3 ng/µl to 1.5 ng/µl and 0.8 ng/µl in A431 cells treated with LDE-225 and Itraconazole, respectively; and 2.5 ng/µl to < 0.8 ng/µl in UM‐SCV‐1A cells treated with LDE-225 or Itraconazole, indicating that Hh pathway inhibition increases the chemo sensitivity of VSCC cell lines to Cisplatin. In marked contrast, this effect was not observed in the Hh-independent cell line, UCI-VULV1 (Fig. 6).

### Hedgehog inhibitors impair cell proliferation and stratification of VSCC cell lines in organotypic raft culture

**Figure 7:**
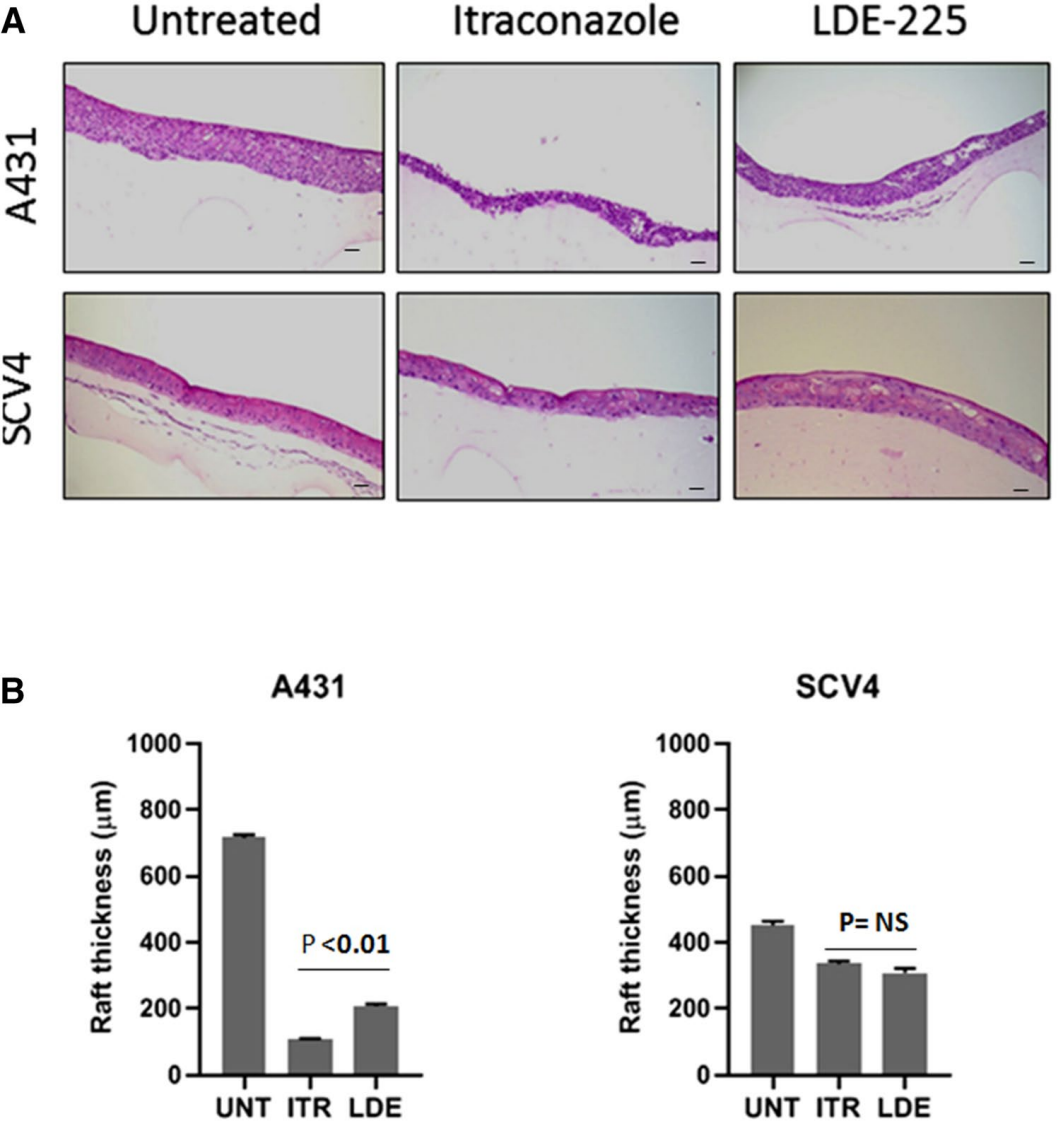

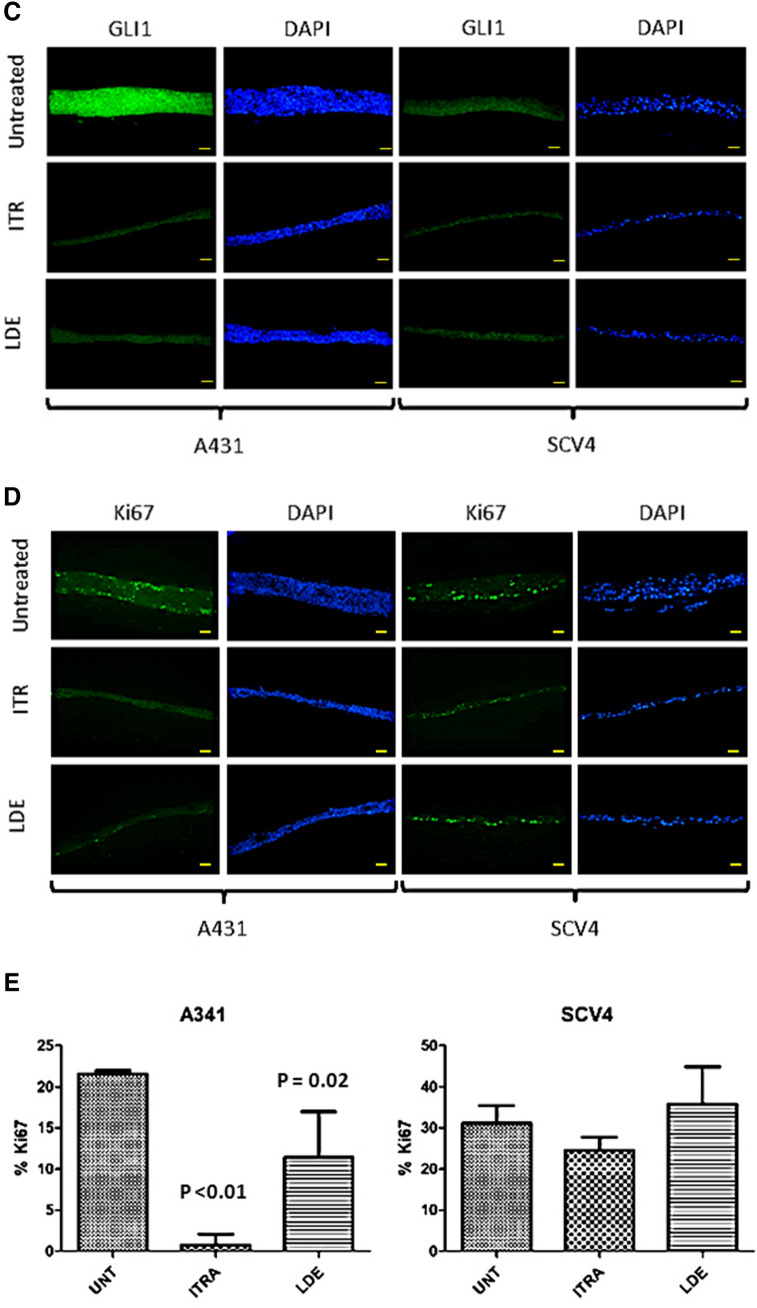
Treatment of Hh-dependent VSCC-derived cell lines in organotypic raft culture with 2.5 µM Itraconazole and 10 µM LDE-225 reduces GLI1 expression and is associated with decreased cell proliferation. (**A**) (upper panels) H&E stained organotypic raft cultures showing a significant reduction in the thickness of epithelium in A431 (Hh-dependent) but not in UM‐SCV‐4 (Hh-independent) following seven days treatment with Itraconazole or LDE-225. (**B**) Graphical output depicting the mean raft thickness in control and treated rafts from A431 and UM-SCV4. Data represent the mean from 6 measurements. NS = none significant. (**C**,**D**) Immunofluorescence staining of control and treated raft culture sections showing reduced expression of GLI1 (**C**) and Ki67 (**D**) in the A431 cell line but not in UM‐SCV‐4 following treatment with 2.5 µM Itraconazole (ITR) or 10 µM LDE-225 (LDE). (**E**) Graphical output depicting the mean number of Ki67 positive nuclei in control and treated rafts from A431 and UM-SCV4. Data represent the mean from 3 measurements from 5 independent raft sections. All experiments were performed at least three times. Bar = 50 μm.

To examine the effects of SMO inhibitors on the growth of VSCC cells in three-dimensional cell culture, organotypic raft cultures were generated from single representative Hh-responsive and non-responsive cell lines, A431 and UM‐SCV‐4. These two cell lines were chosen as the other VSCC-derived cell lines failed to stratify and form differentiating epithelial structures in raft culture (data not shown). After growth at the air: liquid interface for 7 days, rafts were treated with 2.5 µM Itraconazole, 10 µM LDE-225 or DMSO (negative control) for an additional 7 days prior to fixation and processing for histology. Typically, we found that raft cultures generated from the Hh pathway-dependent cell line, A431, formed significantly thinner, less stratified epithelium in response to treatment with either Itraconazole or LDE-225. In contrast, the UM‐SCV‐4 cell line was largely unaffected by treatment with either drug (Fig. 7A,B). Immunofluorescence (IF) staining revealed a significant reduction in nuclear GLI1 expression (Fig. 7C) and reduced staining for the nuclear cell proliferation marker Ki67 (Fig. 7D,E) in response to treatment with SMO inhibitors; in all cases, Itraconazole appeared to be more effective at reducing cell proliferation and GLI1 expression than LDE-225 at the chosen concentrations (Supplementary Fig. 7). Treatment with Itraconazole or LDE-225 on raft cultures generated from Hh-independent cells had little impact on cell growth, GLI1 and Ki67 expression (Fig. 7C,D).

## Discussion

While various genetic and epigenetic alterations have been identified in VSCC, the contribution of dysregulated cell signalling pathways to the aetiology of this cancer is poorly defined. Here we show that VSCC displays frequent activation of the Hh pathway. While Hh pathway components were virtually undetectable in normal squamous vulval epithelium, we found a significant proportion of VSCCs overexpressed SHH ligand, displayed higher levels of nuclear GLI1 and GLI2 proteins, and increased expression of the Hh receptor and putative target gene, *PTCH1*. In our previous study, we found that approximately 90% of VSCC cases over-expressed at least one Hh pathway component, indicating that the Hh pathway may be a primary driver for carcinogenesis in most cases of VSCC^9^. Owing to a lack of tissue material, we were only able to undertake an IHC study on three key Hh pathway components: SHH, PTCH1 and GLI1 in our previous study. In our current study, we secured access to more tissue material which allowed us to undertake IHC staining for additional Hh pathway components and compare their expression with age-matched control vulval epithelium. The results from our recent cohort study re-affirm that the Hh pathway is aberrantly activated in most VSCC, offerings us an opportunity to explore and develop targeted therapies in VSCC for those patients displaying aberrant Hh pathway activation.

A variety of mechanisms can result in aberrant activation of the Hh pathway. While over-expression of SHH ligand is a common mechanism, loss of function mutations in *PTCH1* or activating mutations in *SMO*, can both activate the Hh pathway in a ligand-independent manner^10^. While our study did not directly examine VSCC tumours for evidence of *PTCH1* or *SMO* gene mutations, we observed frequent overexpression of SHH ligand in VSCC specimens^9^. Furthermore, findings from our in vitro studies suggest that SHH ligand overexpression most likely constitutes the mechanism of Hh pathway activation, given that Hh-responsive cell lines were sensitive to either SHH ligand or SMO inhibition or depletion; interestingly, a similar scenario has been described in cervical cancer^11^.

Our study shows that four of six VSCC cell lines displayed high baseline Hh-pathway activity, and that pathway inhibition, either through SHH ligand depletion/antagonism or SMO depletion/inhibition, led to a reduction in GLI1 expression and baseline GLI activity, and a cessation of cell growth. These findings were recapitulated in 3-dimensional organotypic raft culture, where SMO inhibitors inhibited cell proliferation and the formation of stratified epithelial structures. Inhibition of SMO with either siRNA or SMO inhibitors in VSCC cell lines showing no baseline Hh pathway activity had little effect on cell growth. This indicates the potential of developing targeted chemotherapy for VSCC that target SMO and different components of the Hh pathway, which will benefit most patients with VSCC. The fact that the SMO inhibitors, LDE-225 and Itraconazole, were effective at inhibiting the growth of Hh-dependent VSCC cell lines, paves the way for a “window trial” to examine their efficacy in the clinical setting.

Aberrant Hh signalling is linked to chemoresistance in cancer, limiting the effectiveness of chemotherapy^19^. Here, we show that treatment of Hh-dependent VSCC cell lines with two Hh inhibitors sensitises cells to the genotoxic actions of Cisplatin, while Hh-independent VSCC cells neither respond to Cisplatin treatment alone nor Cisplatin co-treatment with Itraconazole or LDE-225. Although the mechanism(s) involved are currently unknown, it may stem from an ability of Hh to regulate expression of anti-apoptotic and drug efflux proteins, hence making cells more resistant to genotoxic drugs^20^. This finding highlights the potential use of Hh inhibitors as adjuvant therapy. Hh inhibitors, in conjunction with lower doses of platinum-based chemotherapy regimens, can be used to treat patients with advanced localised or metastatic disease, minimising chemotherapy-related toxicity. However, it is equally possible for Hh inhibitors to augment the toxicity of chemotherapeutic agents. As the use of Hh inhibitors in clinical practice is relatively recent^21^, early phase clinical trial may be required to assess the toxicity of combination therapy with Hh inhibitor in patients with VSCC. Itraconazole, an anti-fungal drug commonly used to treat refractory vaginal candidiasis, has a relatively low toxicity profile either given through oral or intravenous route^17,22^. As such, it may be a candidate for combination therapy or as a short-term single adjuvant therapy for those who received primary surgery where the tumour is found to exhibit Hh pathway dysregulation.

In conclusion, our study shows that the Hh signalling pathway is frequently activated in VSCC, and that pathway activation is most likely driven by overexpression of SHH ligand. Four of the six VSCC-derived cell lines examined in our study displayed elevated baseline Hh pathway activity, suggesting that pathway dysregulation in the cell lines examined is tumour cell-specific, although it does not rule out the possibility that Hh pathway activation may occur through paracrine mechanisms in vivo. Hh pathway activity appeared necessary to sustain cell growth and viability, as SHH ligand depletion/antagonism or SMO inhibition, either with chemical inhibitors or SMO-specific siRNA, attenuated GLI1 activity and cell proliferation. Although we have yet to establish the mechanism(s) involved in pathway activation in VSCC, the therapeutic effect of both Robotikinin and the two SMO inhibitors, Itraconazole and LDE-225, appeared to be specific to VSCC cells displaying elevated Hh pathway activity. This opens up an opportunity to develop targeted therapies, where routine IHC can quickly determine Hh pathway component expression, and where patients whose tumours display aberrant Hh activation can be offered Hh inhibitors. In our previous study, we found that patients with tumours showing low or negligible levels of the PTCH1 protein, were at increased risk of developing a local recurrence; this group of patients may benefit from adjuvant therapies using Hh inhibitors. Our findings offer an opportunity to explore further and develop a new treatment where the effectiveness of current chemotherapy for the treatment of VSCC remains questionable. Future studies aim not only to explore the therapeutic potential of Hh inhibitors in VSCC but will also focus on determining the molecular defects leading to aberrant Hh activity.

## Materials and methods

### Immunohistochemical (IHC) staining

Archival formalin-fixed paraffin-embedded (FFPE) histology blocks containing primary tumours, along with normal vulval epithelium from age-matched controls, were retrieved and 4 µm sections processed for immunohistochemistry as previously described^23,24^. The antibodies used included antibodies specific for SHH, PTCH1, GLI1, GLI2 and GLI3 (Supplementary Table 1). Over- or under-expression of Hh pathway components in both primary tumours and normal vulval epithelium was determined using the Allred scoring system. Arbitrary values are based on the proportion of positivity and the intensity of staining (Range 0–9)^25^. Histology and staining were comprehensively reviewed by a gynaecological cancer specialist pathologist, RG. Wilcoxon two sample test was used to compare the scores between VSCC and normal vulval epithelium. The use of human tissues (FFPE tissue biopsies) in this study was approved by the National Research Ethics Service Committee West Midlands – Solihull (Reference 11/WM/0070). Archival and historical tissue samples were used in this study and all the experiments were performed in accordance to the relevant guidelines and regulations. Written consent was obtained from patients who gave permission for the FFPE tissues to be used in this study. All study samples were anonymized.

### Cell culture

UCI-VULV-1 cell line was gifted by Dr Carpenter, University of California, US^26^. UM‐SCV‐3, UM‐SCV‐4, UM‐SCV‐1A and UM‐SCV‐1B were gifted by Dr Grénman, University of Turku, Finland^27,28^. All VSCC-derived cell lines were cultured in RPMI1640 supplemented with 10% FBS, 2 mM Glutamine, and antibiotics (penicillin/streptomycin). Normal vulval keratinocytes (NVK) were cultured on an irradiated feeder layer of Swiss 3T3 fibroblasts according to previously published protocols^29^. Short-term cell viability assay: Cells were seeded onto 48-well plates at 5 × 10^3^ cells per well and incubated overnight. Cells were treated with a range of LDE-225 concentrations over 72 h. Cells were fixed with ice-cold 100% methanol and then stained with 1% crystal violet dye. Representative images were taken using bright field microscopy.

### Chemical and pharmacological inhibitors

Itraconazole, LDE-225 and Robotnikinin were purchased from Selleckchem, UK, Sigma-Aldrich, UK and Generon, UK, respectively. Stock solutions were dissolved in DMSO and diluted in complete growth medium just prior to use. XTT assay: Cells were seeded onto 96-well plates at 3 × 10^4^ cells per well and treated with a range of Hh inhibitor concentrations for 72 h. Cell proliferation was then measured by XTT assay according to manufacturer’s instructions (Roche, UK).

### GLI Luciferase reporter assay

Cells were seeded into 12-well plates at 2 × 10^5^ cells and transiently transfected with a GLI luciferase reporter plasmid (pGLIx8) or empty vector control (pGL3) along with a plasmid encoding Renilla luciferase (pRL) using Lipofectamine LTX/Plus reagent in Opti-MEM (ThermoFisher Scientific, UK). 24 h post transfection, cells were switched to complete growth medium containing the drug of interest, and cells incubated for an additional 24 h. Bioluminescence was measured using the Dual-Luciferase Reporter Assay System (Promega, UK) according to manufacturer’s instructions. Data is presented as relative luminescence and is expressed on an arbitrary scale.

### Western blotting

Cell protein lysates were resolved by SDS-PAGE, transferred onto Hydrobond nitrocellulose membranes (VWR) and probed with primary antibodies overnight. Blots were incubated with HRP-conjugated secondary antibodies (Dako, UK). Proteins were visualised by chemiluminescence (Geneflow, UK), using the Fusion FX Spectra (Vilber, UK) gel doc system. Densitometric analysis using ImageJ provided semi-quantitative results.

### siRNA gene knockdown

Cells were plated into 6 well plates at a density of 3 × 10^5^ cells per well or 3 × 10^4^ per well in 96 well plates and grown until approximately 80% confluent. Cells were transiently transfected with either a SMO Stealth RNAi siRNA (HSS185995 and HSS185994) or Stealth RNAi siRNA Negative Control Lo GC Duplex (ThermoFisher Scientific, UK), using Lipofectamine RNAiMAX (Invitrogen, UK) according to manufacturer’s instructions. Transfected cells were either lysed to generate protein lysates or plated for an XTT assay. For co-transfection, cells were plated into 6 well plates and grown until 80% confluent. Cells were co-transfected with either a SMO Stealth RNAi siRNA (or negative control siRNA (ThermoFisher Scientific, UK), along with the GLI -luciferase reporter plasmid (pGLIx8) or empty vector control (pGL3) to perform GLI -luciferase reporter assays. Transfection of the siRNA complexes into the cells were performed using the Lipofectamine RNAiMAX protocol (Invitrogen, UK). Cells were simultaneously transfected with the control or GLI-reporter plasmids using Lipofectamine LTX with Plus reagent (ThermoFisher Scientific, UK). GLI reporter assays were performed 24–48-h post-transfection.

### Quantitative polymerase chain reaction (qPCR)

SHH, PTCH SMO, GLI1, GLI2, GLI3, SUFU, BMI1 and FOXM1 mRNA expression was determined by TaqMan qPCR assay. Sequence-specific primers and Fluorescein-labelled probes designed for GAPDH and gene-specific primers were purchased from ABI Systems (ThermoFisher Scientific, UK) (Supplementary Table 2). Total DNA was extracted from cell lines using DNeasy kit (Qiagen, UK) according to manufacturer’s protocol, and 200 ng genomic DNA was amplified using FastStart PCR Master mastermix (Sigma-Aldrich, UK), PCR primers with ABI 7700 Sequence Detection System. PCR conditions were: initial enzyme activation step (50 °C/2 min), a denaturation step at (95 °C/10 min), followed by 50 cycles of denaturation (95 °C/15 s) and annealing/extension step (55 °C/1 min). Normal vulval keratinocytes (NVK) were included as controls.

### Organotypic raft cultures

Rafts were prepared according to the protocol described previously^30^. Cells were seeded onto a collagen lattice containing 3T3-J2 fibroblasts and grown until confluent. The collagen lattice was transferred to a stainless-steel grid and exposed at the air/liquid interface 2 weeks. Rafts were treated with either 2.5 µM Itraconazole, 10 µM LDE-225 or DMSO (control) every 2 days in the second week of the air/liquid interface. Rafts were fixed with 4% paraformaldehyde in DMEM and processed for immunostaining.

### Immunofluorescence staining

Raft sections were processed for imaging as described previously^31^. Samples were incubated with primary antibodies followed by secondary antibodies in 20% HINGS. The antibodies used are listed in Supplementary Table 1. Slides were mounted in fluoroshield (Sigma, UK) containing DAPI (Invitrogen, UK). Images were obtained at 200X magnification on a fluorescence microscope.

### Statistical analysis

Statistical analyses were performed using GraphPad Prism version 8.0.1. Categorical variables were analysed using the student t-test. A *p*-value of less than 0.05 was regarded as statistically significant.

## Supplementary Information

Supplementary Information.

## Data Availability

The datasets generated during and/or analysed during the current study are available from the corresponding author on reasonable request.
