# Supplementary material for: Aberrant activation of the Hedgehog signalling pathway in squamous cell carcinoma of the vulva as a potential target for cancer therapy
Source: Sci Rep. 2021 Sep 3;11:17665. doi: 10.1038/s41598-021-96940-1 (PMC8417215; doi:10.1038/s41598-021-96940-1)

**Aberrant activation of the Hedgehog Signalling pathway in squamous cell carcinoma of the vulva as a potential target for cancer therapy**

Jason Yap^*1,2^, Khalil Uddin^1^, Rachel Pounds^1,2^, Danielle O’Neill^1^, Sean Kehoe^1,2^, Raji Ganesan^3^, Christopher Dawson^4*^

^1^Birmingham Cancer Research UK Cancer Centre, Institute of Cancer and Genomic Sciences, University of Birmingham, Birmingham, B15 2TT, UK.

^2^Pan Birmingham Gynaecological Cancer Centre, City Hospital, Dudley Road B18 7QH, UK.

^3^Birmingham Women’s NHS Foundation Trust, Mindelsohn Way, Edgbaston, Birmingham B15 2TG, UK.

^4^Department of Microbiology & Infection, Warwick Medical School, University of Warwick, CV4 7AL.

*Corresponding authors

**
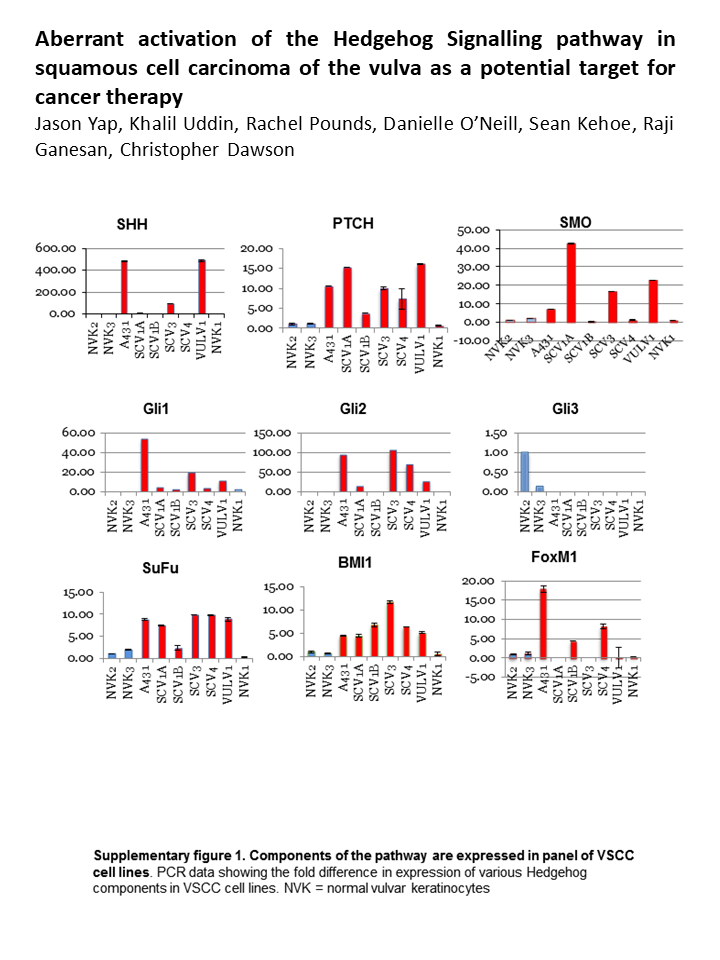
**

**Supplementary Figure 1.** Quantitative PCR data showing the fold difference in expression of various Hedgehog components and target genes in VSCC cell lines and normal vulval keratinocytes. NVK = normal vulval keratinocytes.

**
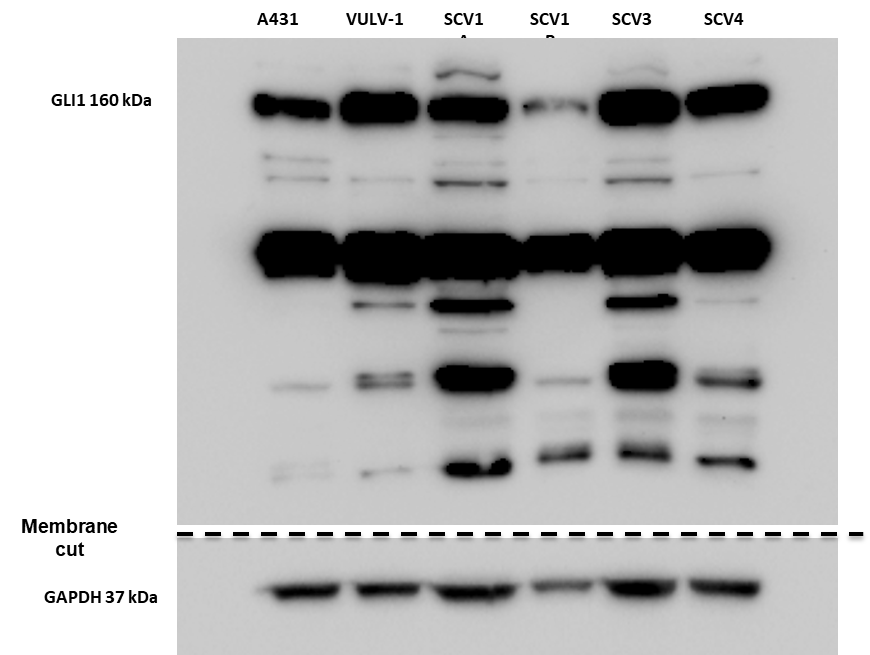
**

**GLI1**

**GLI2**

**
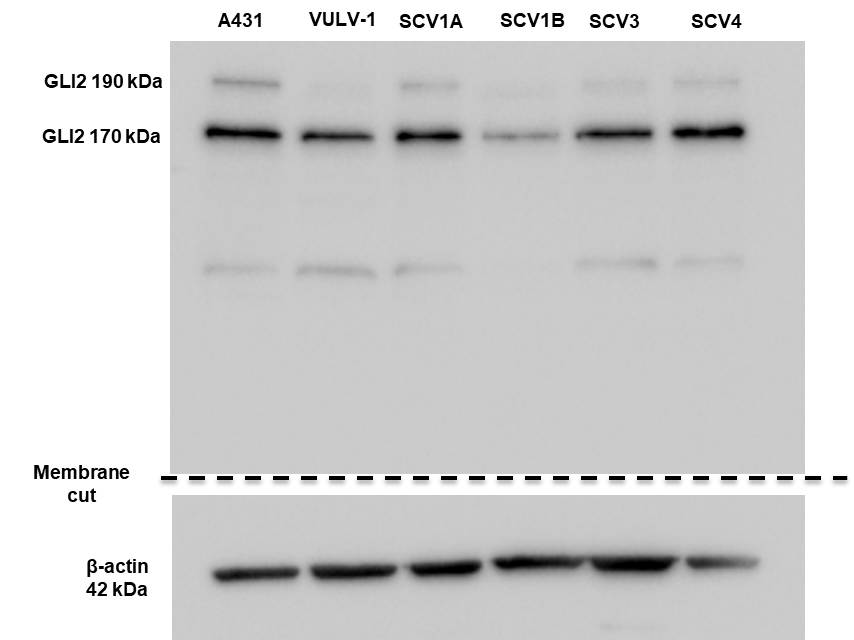
**

**Supplementary Figure 2**. Western blot analysis confirming expression GLI1 and GLI2 proteins in all of the VSCC cell lines. All experiments were performed 3 times.


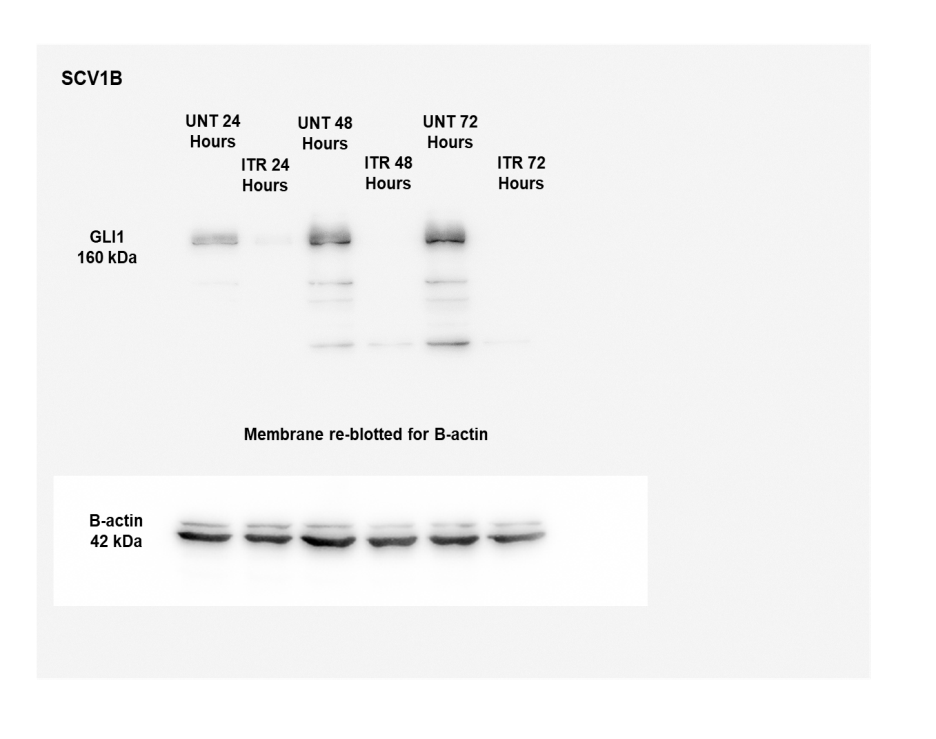

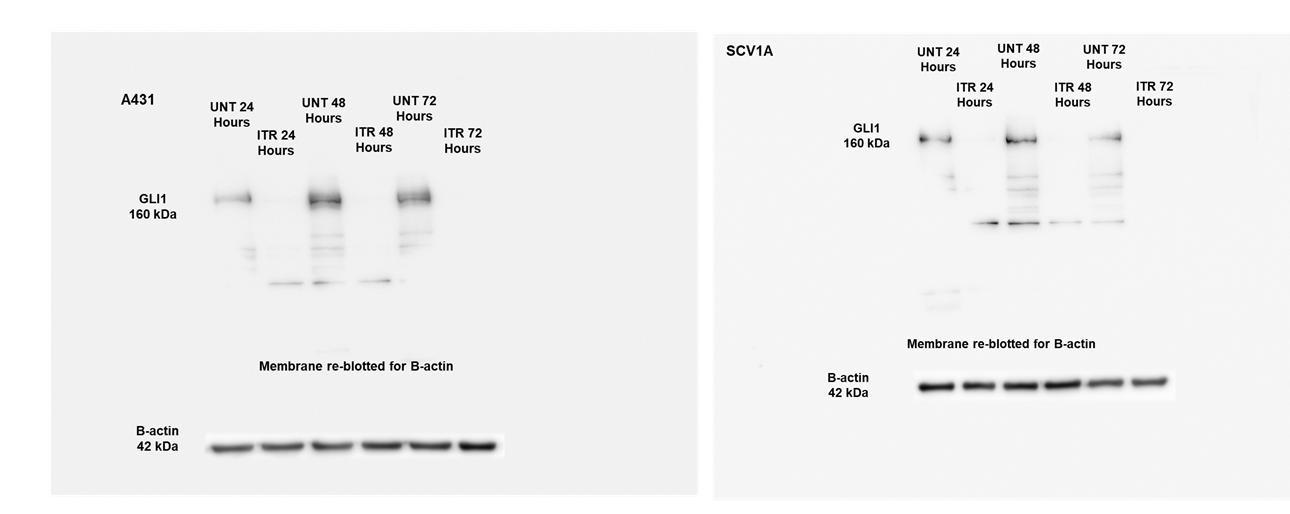


**A.**

**B.**

**B.**


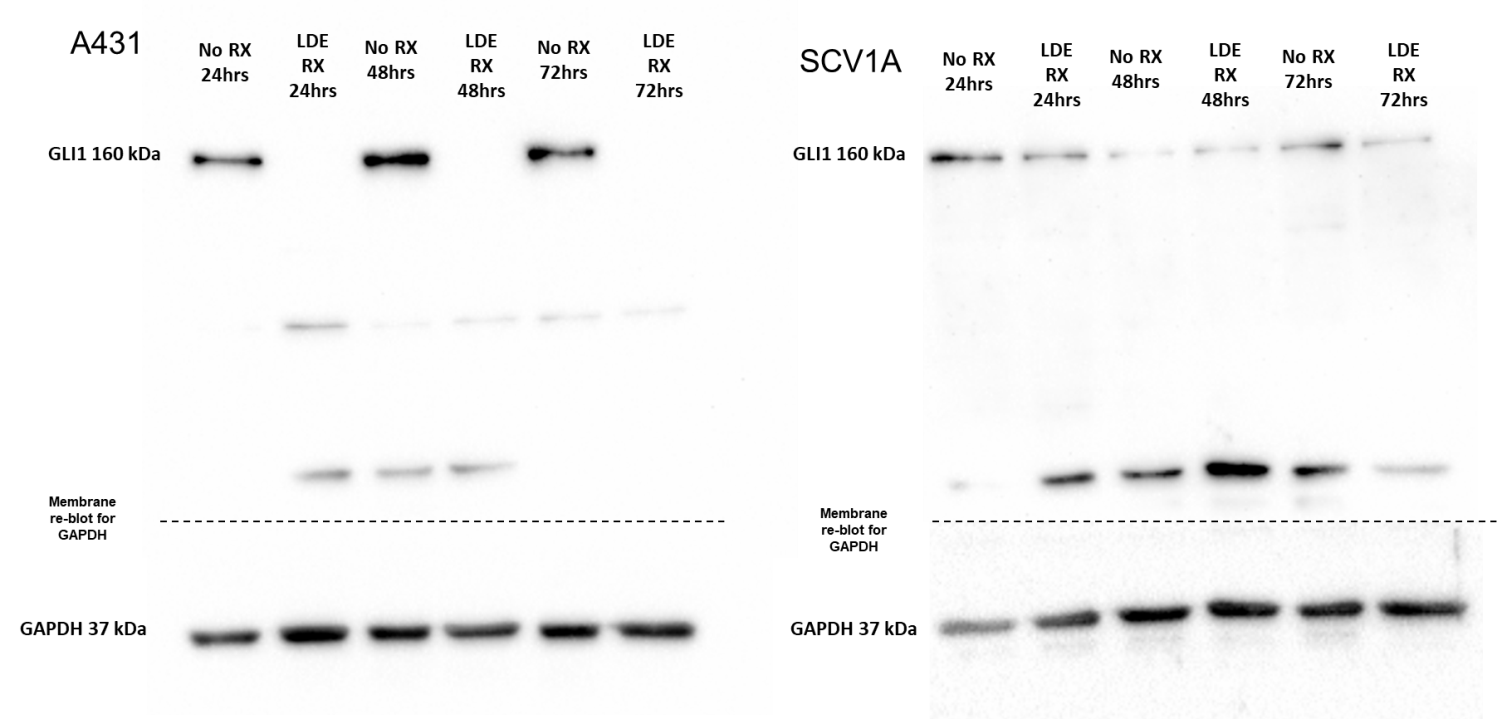


**Supplementary Figure 3.** (A) Uncropped Western blot showing the reduction in GLI1 expression in Hh-responsive cells (A431, UM-SCV1A, UM-SCV1B) treated with 10µM Itraconazole (Figure 4C.1) for the indicated times. (B) Uncropped Western blot showing the reduction in GLI1 expression in Hh-responsive cells (A431, UM-SCV1A, UM-SCV1B) treated with 25µM LDE-225 (Figure 4C.2) for the indicated times (UNT= untreated; Rx= treatment).

**
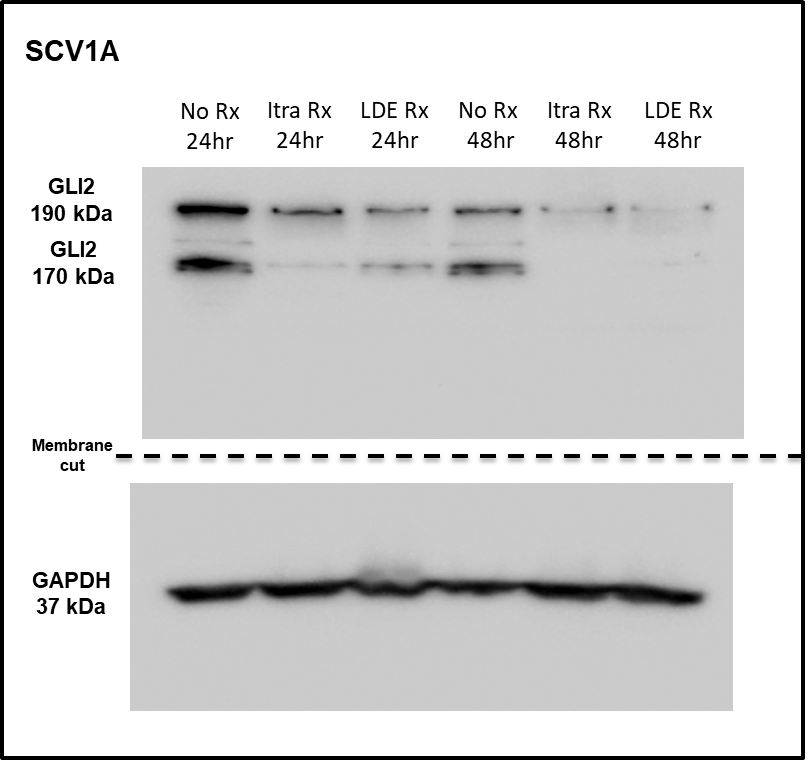
**

**
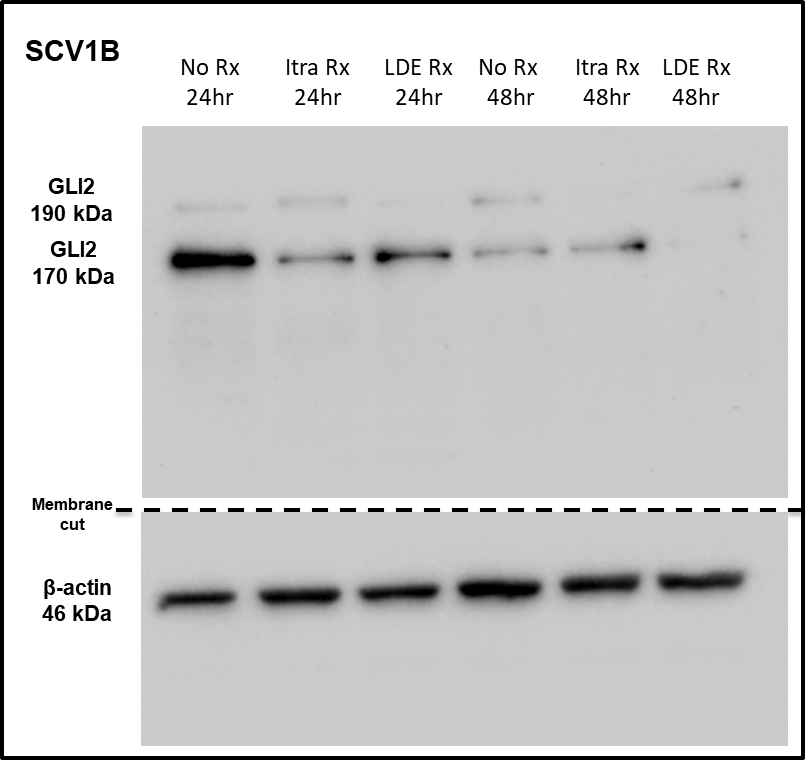
**

**
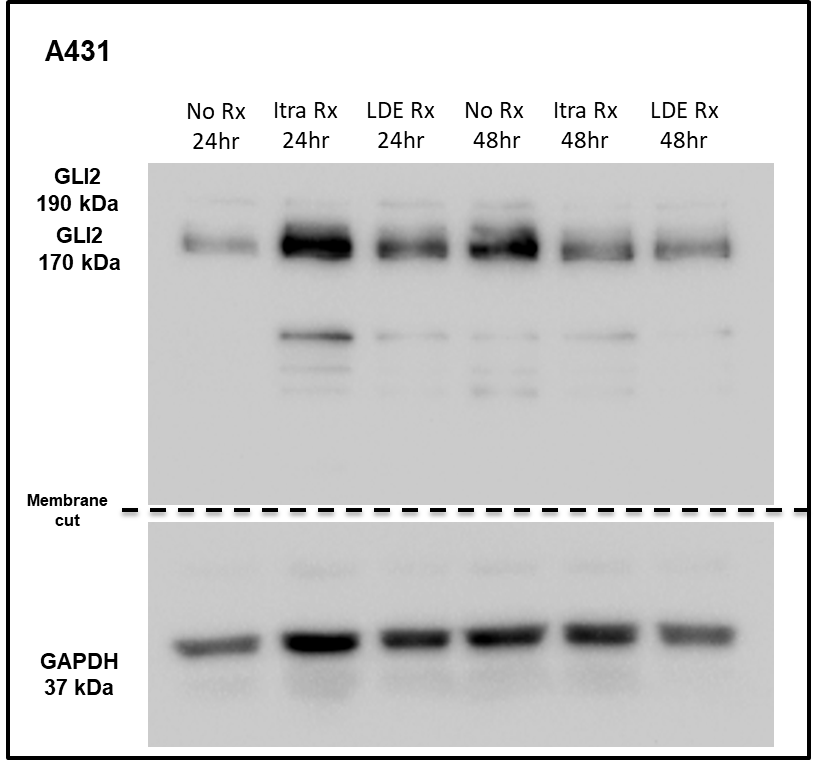
**

**Supplementary Figure 4.** Western blot showing the reduction in GLI2 expression in Hh-responsive cells (UM-SCV1A, UM-SCV1B and A431 cell lines) treated with 10µM Itraconazole and 25µM LDE-225 for 24hrs or 48 hours, respectively (Rx= treatment).

**
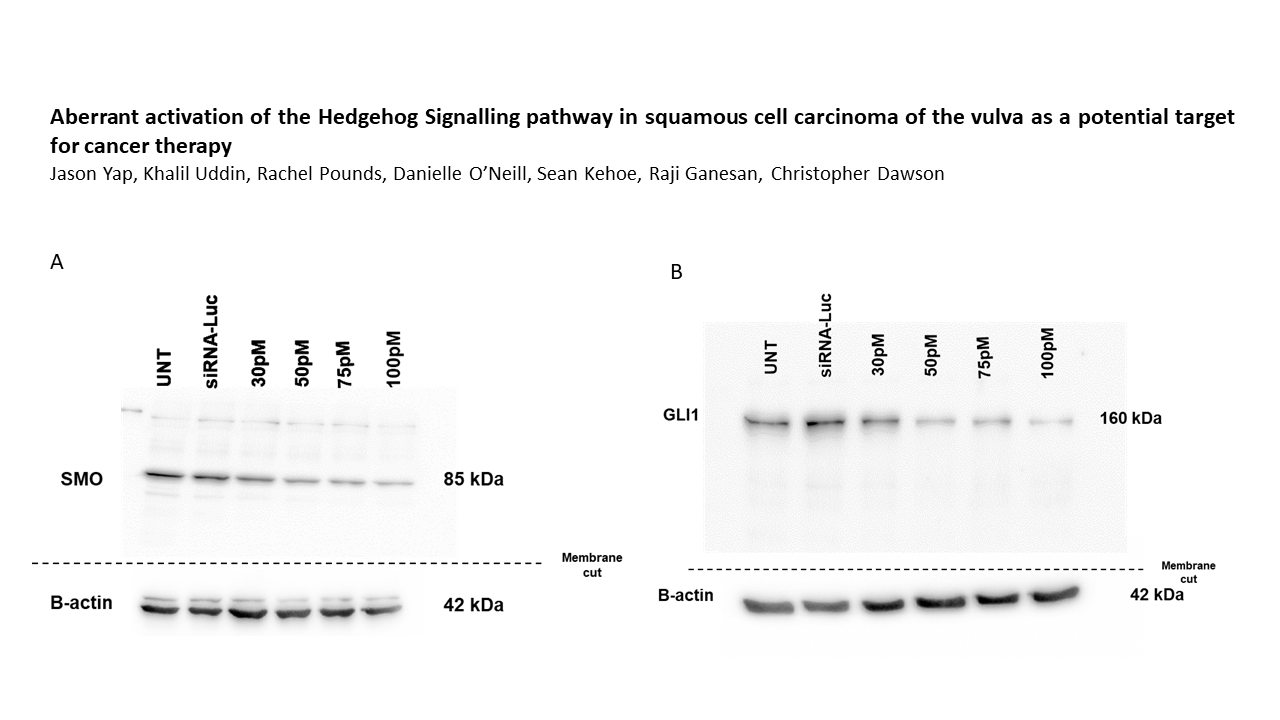
**

**Supplementary Figure 5.** Uncropped Western blot of Figure 5. (A) SMO protein expression, (B) GLI1 protein expression (UNT= untreated).


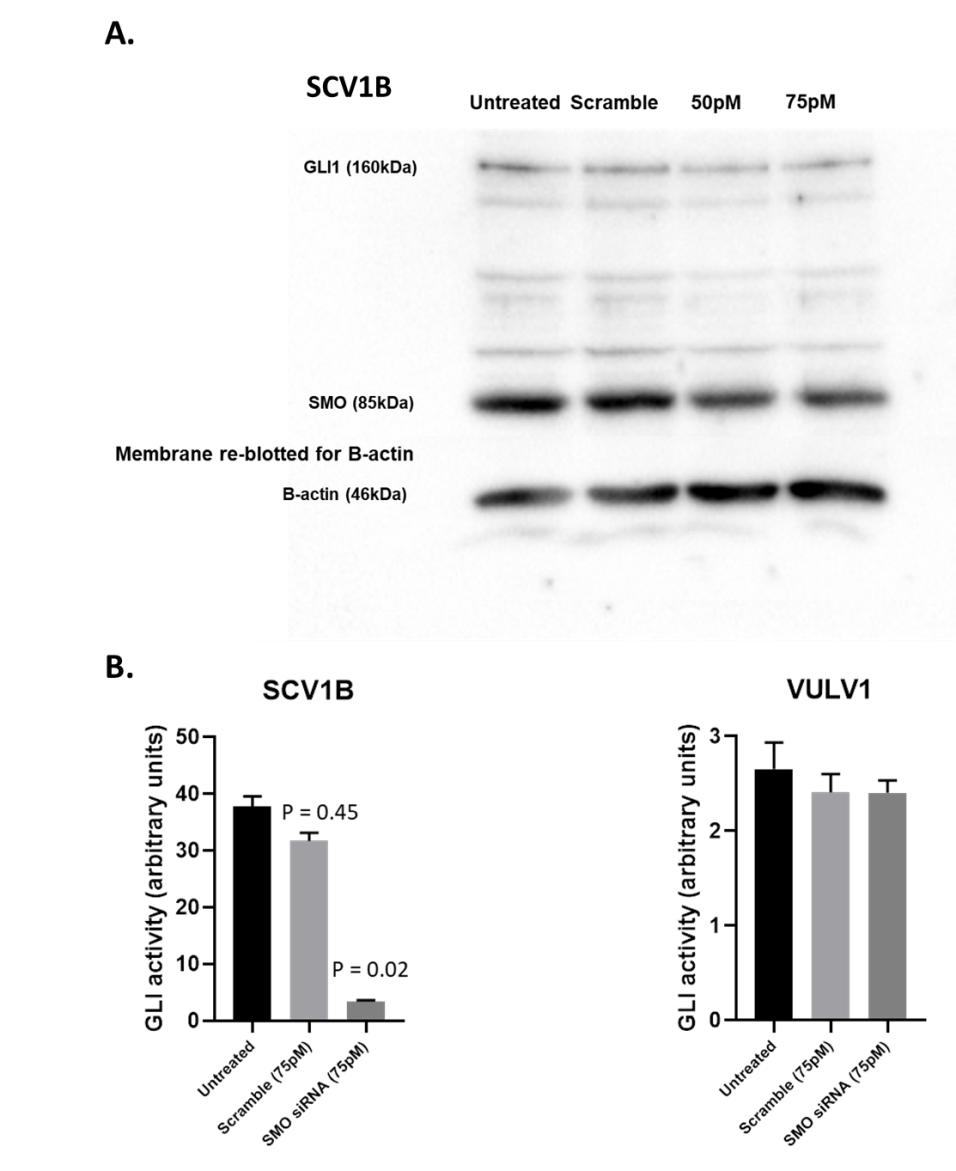


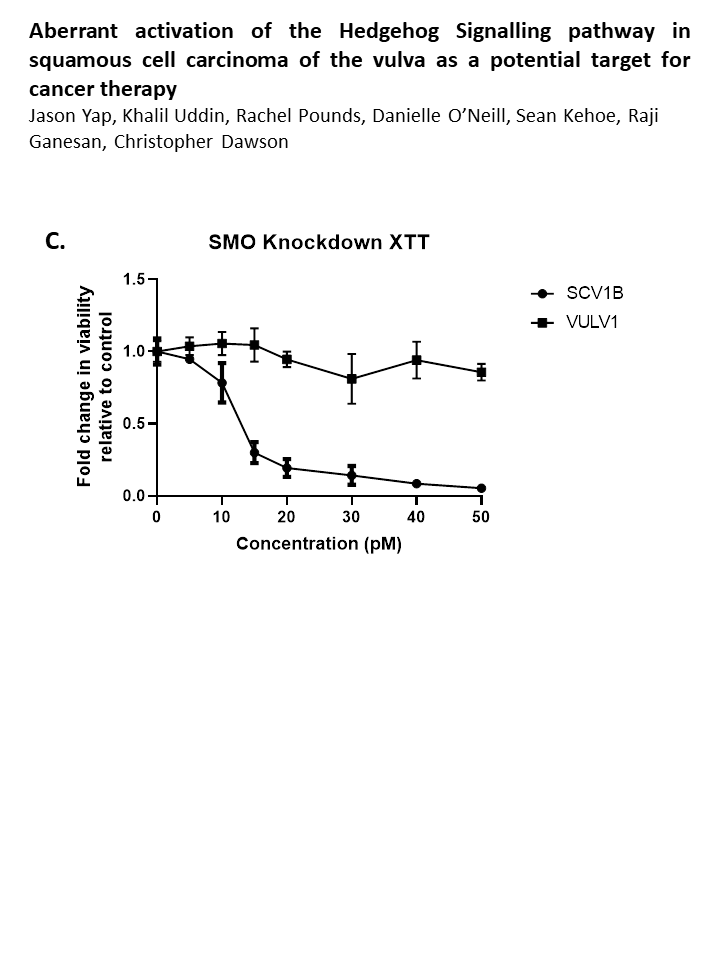


**Supplementary Figure 6.** Transient transfection of Hh-responsive VSCC cell line, UM‐SCV-1B, with a different SMO-specific siRNA (HSS185994), reduces GLI1 activity and cell proliferation. (A) Western blotting analysis showing downregulation of SMO protein and a concomitant reduction in the expression of GLI1, following transfection with SMO-specific siRNAs. No effect on the expression of SMO or GLI1 was observed with a control “scrambled” siRNA. (B) GLI-luciferase reporter assays demonstrating the ability of two independent SMO-specific siRNAs to attenuates Hh/GLI signalling in the Hh-dependent UM-SCV-1B cell line but not the Hh-independent UCI-VULV1 cell line. (C) Unlike UCI-VULV1, transient transfection of a SMO-siRNA in the Hh-responsive UM‐SCV‐1B cell line resulted in a dose-dependent reduction in cell viability. Samples for WB were derived from the same experiment and gels/blots were processed in parallel. All experiments were performed three times.


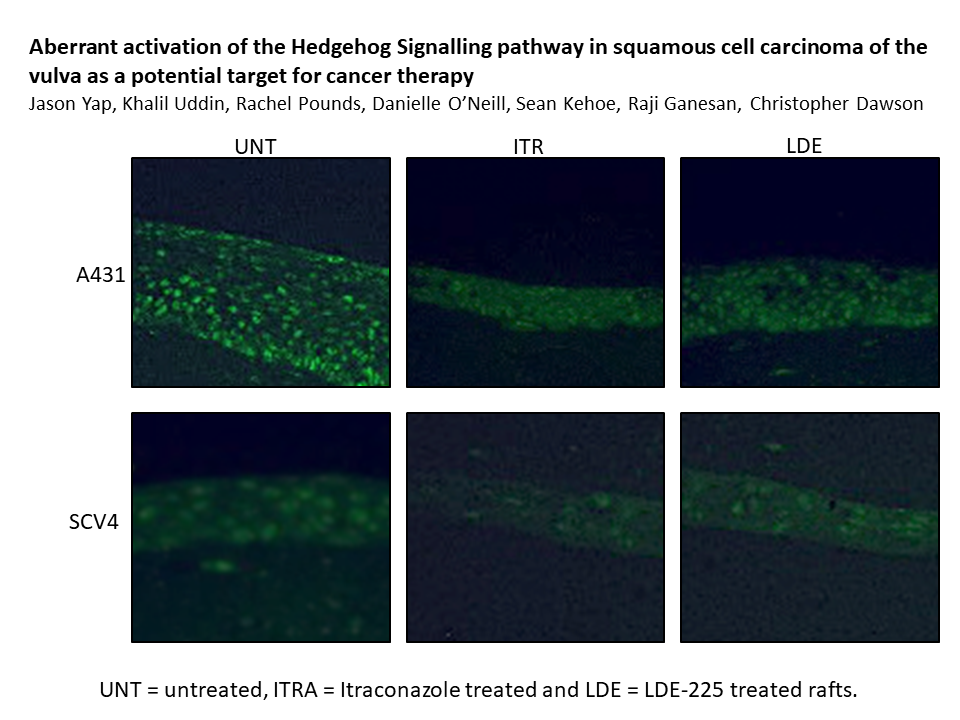


**Supplementary Figure 7.** Higher magnification of Figure 7C (1000x magnification) showing qualitative. Bar = 50μm


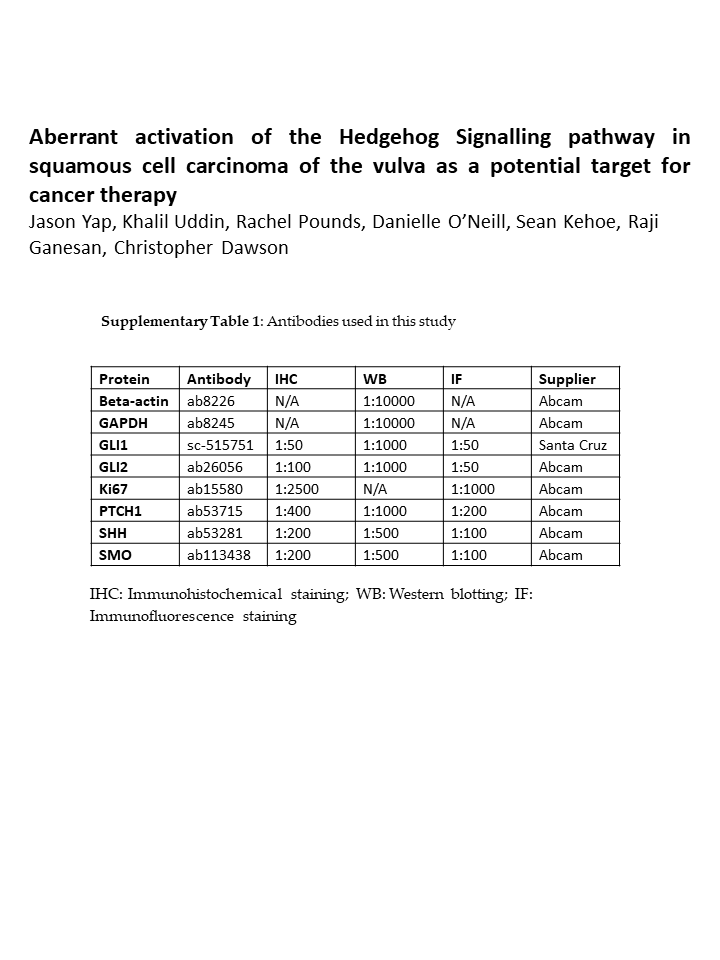


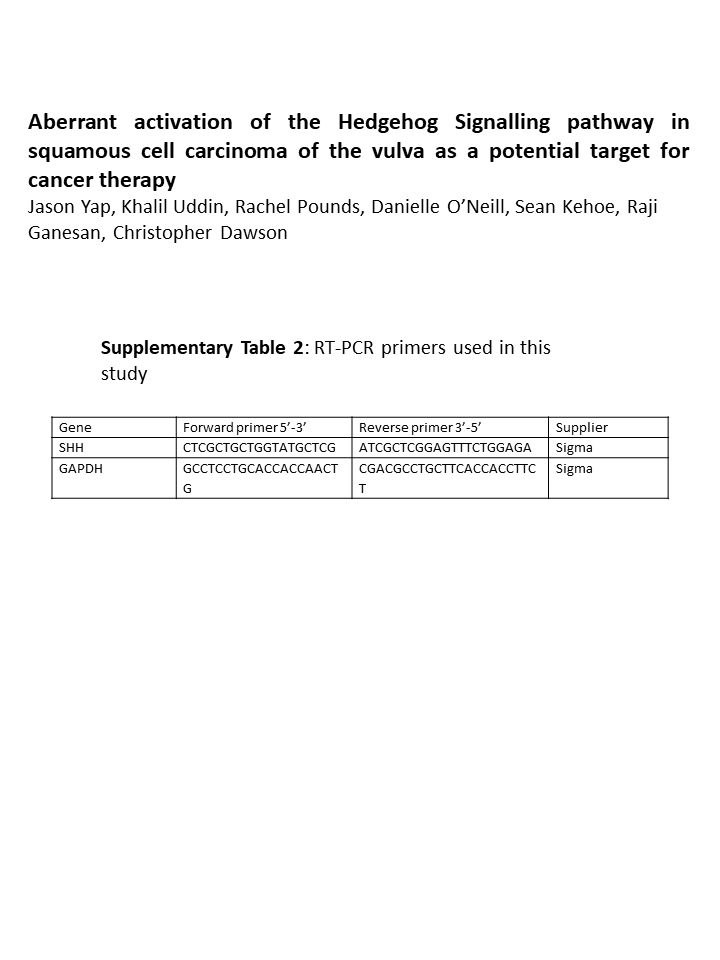

Supplement: Supplementary file 1 — Supplementary Information. [file 41598_2021_96940_MOESM1_ESM.docx]
